# Marine-Derived Compounds Targeting Topoisomerase II in Cancer Cells: A Review

**DOI:** 10.3390/md20110674

**Published:** 2022-10-27

**Authors:** Giulia Greco, Valentina Pellicioni, Ivan Cruz-Chamorro, Giuseppe Attisani, Claudio Stefanelli, Carmela Fimognari

**Affiliations:** 1 Department for Life Quality Studies, University of Bologna, Corso d’Augusto 237, 47921 Rimini, Italy; 2 Departamento de Bioquímica Médica y Biología Molecular e Inmunología, Universidad de Sevilla, 41009 Seville, Spain; 3 Instituto de Biomedicina de Sevilla, IBiS (Universidad de Sevilla, HUVR, Junta de Andalucía, CSIC), 41013 Seville, Spain

**Keywords:** topoisomerase II, cancer chemotherapy, marine compounds, sponges, marine fungi, marine bacteria, marine invertebrates

## Abstract

Cancer affects more than 19 million people and is the second leading cause of death in the world. One of the principal strategies used in cancer therapy is the inhibition of topoisomerase II, involved in the survival of cells. Side effects and adverse reactions limit the use of topoisomerase II inhibitors; hence, research is focused on discovering novel compounds that can inhibit topoisomerase II and have a safer toxicological profile. Marine organisms are a source of secondary metabolites with different pharmacological properties including anticancer activity. The objective of this review is to present and discuss the pharmacological potential of marine-derived compounds whose antitumor activity is mediated by topoisomerase II inhibition. Several compounds derived from sponges, fungi, bacteria, ascidians, and other marine sources have been demonstrated to inhibit topoisomerase II. However, some studies only report docking interactions, whereas others do not fully explain the mechanisms of topoisomerase II inhibition. Further in vitro and in vivo studies are needed, as well as a careful toxicological profile evaluation with a focus on cancer cell selectivity.

## 1. Introduction

Cancer is the second leading cause of death in the world after cardiovascular diseases, affecting an estimated 19 million people and causing approximately 10 million deaths in 2020 [1].

Chemotherapy represents the main anticancer therapeutic approach. Nowadays, the principal clinically employed anticancer drugs are natural products, or their structural analogs [2,3,4,5,6]. However, several factors limit their effectiveness: (i) their efficacy is inversely proportional to disease progression; (ii) occurrence of chemoresistance; (iii) severe toxicity caused by lack of selectivity against cancer cells [7,8]. For this reason, the discovery of anticancer agents characterized by an improved pharmaco-toxicological profile remains a major aim of pharmacological research.

One of the principal targets of drugs used in chemotherapy to stop the aberrant proliferation of cancer cells is topoisomerase (topo) II [9].

Topo is a class of nuclear enzymes essential for cell survival. They regulate the topology of DNA and are involved in replication, transcription, proliferation, and chromosome segregation during the cell cycle. Vertebrates express two different isoforms of topo II–α and β–and although they possess 70% sequence homology and show similar enzyme activity, they are expressed and regulated differently [10].

The mechanism of action of topo II is the temporary break of both DNA strands to allow supercoil relaxation and the physiological cellular process.

**Figure 1:**
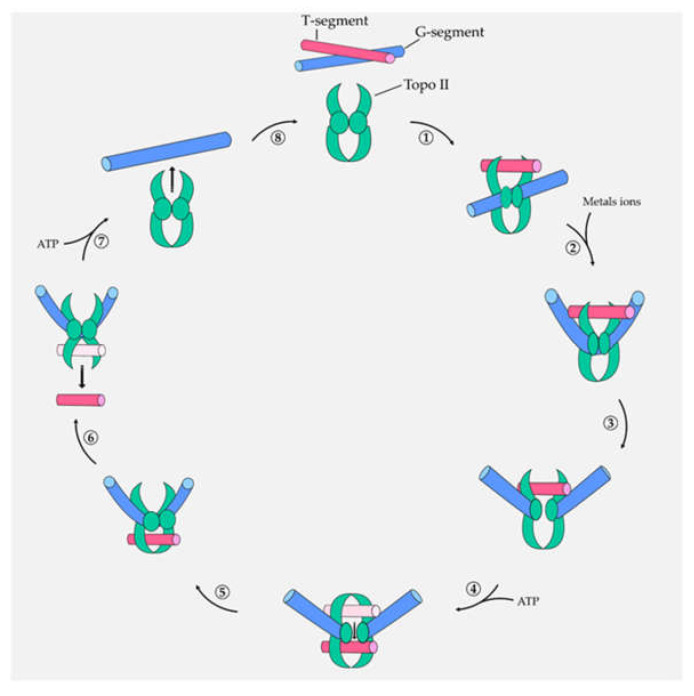
Catalytic cycle of topo II. Binding DNA segments ➀; flexing of the G-segment in the presence of metals ions ➁; formation of the cleavage complex ➂; closing the gate to constrain the T-segment to pass through the G-segment ➃; ligation of the G-segment ➄; release of the T-segment ➅; release of the G-segment ➆; enzyme ready for a new catalytic cycle ➇.

Specifically, topo II acts on both strands of DNA, being capable of removing knots or tangles from the entire DNA duplex. In fact, the cut that is occasioned in a specific region of DNA (Gate-segment) allows another DNA duplex (Transport-segment) to be crossed throughout this break, unwinding the DNA. Topo II generates a covalent interaction–called cleavage complex–with the newly cut G-segment [9]. In particular, the catalytic cycle of topo II is composed of: (i) binding DNA segments (G- and T-); (ii) flexing of the G-segment in the presence of metals ions; (iii) formation of the cleavage complex by a nucleophilic attack occasioned by tyrosine residues present in the catalytic site of the enzyme; (iv) closing the gate to constrain the T-segment to pass through G-segment; (v) ligation of the G-segment and release of the T-segment, and (vi) releasing of the G-segment mediated by ATP hydrolysis and arrangement of the enzyme for a new catalytic cycle (Figure 1) [9]. 

Thus, the inhibition of topo activity allows the blocking of the cell cycle and then conduces to cell death [11]. Topo II-mediated DNA breakage is a critical step for cell survival and must be finely regulated to avoid a possible fragmentation of the entire genome [9]. In a healthy cell, there is fine control of the formation of cleavage complexes, which are short-lived and reversible. Topo II inhibitors are compounds capable of modulating the formation of cleavable complexes and altering this equilibrium. 

There are two different mechanisms described for topo II inhibition: (i) poisoning or (ii) catalytic inhibition. Poisoning is the main mechanism and acts on the stabilization of the cleavable complex, leading to maintaining the permanent breakage of DNA. Indeed, when the levels of cleavable complexes become high, they cannot be repaired by topo II, thus becoming irreversible DNA lesions that activate different signaling pathways and result in cell death by apoptosis [12]. On the other hand, catalytic inhibition implies that the inhibitor prevents the formation of the cleavage complex. If the amount of cleavage complexes is poor, the DNA relaxation is impeded, the daughter chromosomes remain entangled, and segregation is not possible during mitotic replication. In addition, in this case, apoptotic cell death is activated [9]. 

The stabilization of the cleavage complex mediated by topo II poisons or the blocking of its catalytic activity by topo II catalytic inhibitors are two opposite processes that both lead to cell death by induction of apoptosis.

One of the most important classes of anticancer drugs targeting topo II is anthracyclines, extracted from *Streptomyces* genus bacteria. The most used anthracycline is doxorubicin (DOXO), as well as its epimer epirubicin [13] and its derived valrubicin [13] that act as topo II poisons [14]. However, it has been described that the use of DOXO leads to important side effects, such as cardiomyopathy [15].

Other drugs, derived from a natural source, act as topo II poisons: for instance, etoposide (ETO) and its analog teniposide, two podophyllotoxins obtained from the herbaceous plant of the *Podophyllum* genus [16], and resveratrol [17], an important polyphenol found in several vegetable sources. Examples of natural topo II catalytic inhibitors are tryptanthrin, obtained from *Candida lipolytica* yeast or from several plant genera as *Clanthe*, *Isatis*, *Wrightia*, *Couroupota* [18]; fisetin [19] and myricetin [20], flavonoids present in several fruits; or daurinol [21], a triterpene isolated from *Haplophyllum dauricum*.

For several years, the research of new molecules with anticancer activity has also been centered on marine sources such as sponges, fungi, bacteria, etc. [22]. In fact, due to some unfavorable environmental conditions, such as salinity, temperature and pressure alterations, competition for free soil, etc., the marine organisms had to develop several adaptative mechanisms, which are mediated by the secondary metabolites [23]. Secondary metabolites exhibit a wide range of biological effects and pharmaceutical activities. However, their discovery and characterization are limited by the low quantities that are achievable from these organisms. Despite this limitation, several marine-derived compounds possess very interesting antitumor potential [24].

The purpose of this study was to investigate compounds derived from marine sources–specifically sponges, fungi, bacteria, ascidians, echinoderms, and marine microalgae, with proven antitumor activity mediated by topo II inhibition–through a systematic review. In July 2022, a literature search was conducted using the public databases Pubmed Scopus, and Web of Science. The search strategy used free descriptors and terms, limiting articles to the human topo II and the English language, regardless of publication year. The search retrieved all relevant articles related to marine-derived compounds, topo II inhibition, and antitumor activity. In vivo and in vitro studies are included. 

## 2. Topo II Inhibitors from Marine Sponges

### 2.1. Neoamphimedine and Amphimedine

**Figure 2:**
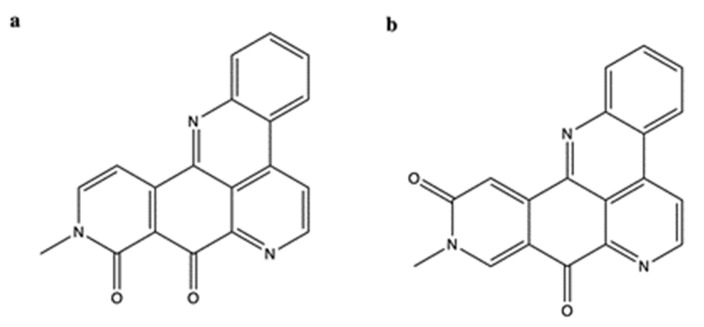
Chemical structure of neoamphimedine ((**a**), CAS number: 221456-55-9) and amphimedine ((**b**), CAS number: 86047-14-5).

The alkaloids neoamphimedine (**neo**) (Figure 2a) and its regioisomer amphimedine (Figure 2b) are two pyridoacridines isolated from *Xestospongia* sp.

**Neo** was highly cytotoxic in several tumor cell lines [25,26]. In addition, **neo** was equally cytotoxic in wild-type A2780 ovarian cancer cells and in multidrug-resistant (MDR)-expressing A2780AD cell line (Table 1). Of note, taxol, DOXO, and amsacrine (m-AMSA) had a 15-, 33-, and 8-fold lower cytotoxicity than neo [25]. In vivo, the administration of **neo** (12.5–50 mg/kg for 19 days) to Balb/c nu/nu mice bearing HCT-116 and KB xenograft reduced tumor growth (Table 1) and displayed the same efficacy as ETO [25].

Focusing on **neo** as a topo II inhibitor, early studies showed that this metabolite did not act as a topo II poison. Firstly, **neo** slightly cleaved DNA through the formation of cleavage complexes (Table 1) [25,27]; secondly, it did not cause a more pronounced cytotoxicity on Chinese hamster ovary (CHO) xrs-6 cells [double strand breaks (DSBs) repair-deficient] compared to CHO AA8 cells (DSBs repair-competent) [25], a typical behavior of topo II poisons [28]. Instead, **neo** inhibited the catalytic activity of topo II through the topo II-mediated catenation of DNA (both supercoiled and relaxed), only in the presence of active topo II, and promoted the aggregation of DNA into high molecular weight complexes, resulting from neither protein–DNA aggregation nor chemical cross-linking to DNA [25,27]. The regioisomer amphimedine did not exhibit any of these effects [25,27]. More recently, Ponder et al. reported that **neo** (0.5–30 μM) suppressed topo IIα-mediated DNA decatenation and competitively inhibited topo IIα-dependent ATP hydrolysis by binding to the ATPase site of the enzyme, with a binding energy equal to −61.8 kcal/mol, as reported in computational docking studies [29]. This in silico approach also elucidated that the lack of activity of amphidemine was due to the different position of the carbonyl moiety, which prevented the binding to the ATPase site of the enzyme. In the same study, the authors analyzed the topo IIα inhibitory activity of **neo** in the presence of metnase, a DNA repair protein that facilitates the reaction of DNA decatenation and contributes to the development of resistance against topo II inhibitors such as DOXO and ETO [30,31]. **Neo** maintained and exhibited even greater topo IIα inhibitory activity, as observed in human embryonic kidney 293 (HEK293) cells that over-express metnase compared to wild-type HEK293 cells [29]. Those results suggest that (i) the binding affinity of neo for topo IIα is higher when topo IIα interacts with metnase, and (ii) neo seems to elude the metnase-based mechanism of resistance. 

The **neo** capability to act as a topo IIα ATP-competitive inhibitor was associated with the reversion of the epithelial–mesenchymal transition (EMT), as shown in a multicellular tumor spheroid model (MCTS) of colon cancer cells (SW620) [32]. EMT is a process that occurs when epithelial cells lose their characteristics and assume a mesenchymal phenotype. EMT boosts the metastatic potential of tumor cells, enabling them to get through the extracellular matrix, get into the bloodstream, and then proliferate in a distinct tissue [33]. Aberrant T-cell factor (TCF) transcription and β-catenin are involved in the Wnt signaling pathway, which actively participates in the EMT process. Topo IIα has a key role in the Wnt signaling pathway: it interacts with β-catenin, TCF4, Wnt response elements (WREs), and promoters of downstream target genes of TCF (c-Myc, vimentin, and axis inhibition protein 2). Acting as a topo IIα ATP-competitive inhibitor, **neo** reduced the topo II-dependent TCF transcription, both in vitro (colorectal cancer MCTS cells; 10 μM, 72 h of treatment) and in vivo (SW620 xenografted athymic nude mice; 5 mg/kg, once a week for 22 days) [32]. In SW620 MCTS, **neo** also prevented the binding of topo IIα and TCF4 to WREs and promoters and reverted EMT through (i) the downregulation of the protein expression of mesenchymal markers (vimentin, Slug, zinc-finger E-box binding homeobox 1, and c-Myc) and (ii) the upregulation of epithelial ones (zonula occludens-1 and E-cadherin) [32]. Overall, **neo**, as a topo IIα inhibitor, downregulates the transcriptional activity of the β-catenin/TCF4 nuclear complex, which can be considered an interesting target for the types of cancers–such as colon cancer–in which the Wnt pathway largely contributes to the carcinogenic process [34].

### 2.2. Aeroplysinin-1 and Its Oxidized Derivative

**Figure 3:**
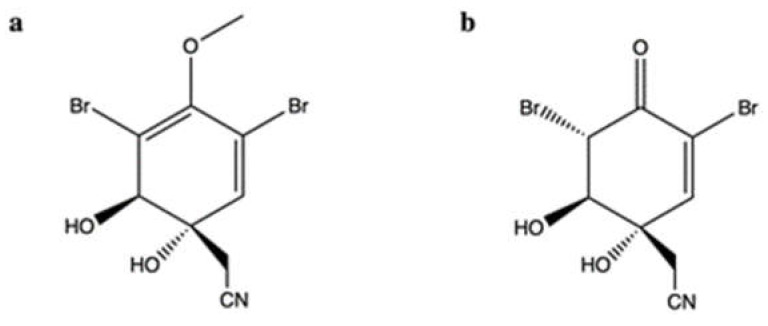
Chemical structure of aeroplysinin-1 ((**a**), CAS number: 28656-91-9) and its oxidized derivative ((**b**), CAS number: 294208-35-8).

The brominated isoxazoline alkaloid aeroplysinin-1 (**apl-1**) (Figure 3a) and its oxidized derivative [**DT**; (1′R,5′S,6′S)-2-(3′,5′-dibromo-1′,6′-dihydroxy-4′-oxocyclohex-2′-enyl) acetonitrile] (Figure 3b) were isolated from the marine sponge *Pseudoceratina* sp. [35,36].

**DT** was cytotoxic on different tumor cell lines. Additionally, **DT** had a selective cytotoxic effect on tumor cells, since the cell viability of rat alveolar macrophage NR8383 cells was more than 80% after exposure to the highest tested concentration of the compound [35]. In the same study, **DT** (0.01–10 μg/mL) was found to inhibit topo IIα using a cell-free DNA cleavage assay with an enzyme-mediated negatively supercoiled pHOT1 plasmid DNA. In the presence of topo IIα, **DT** at low concentrations (0.01, 0.1, and 1 µg/mL) caused DNA relaxation, and at high concentrations (2.5, 5, and 10 µg/mL) blocked DNA relaxation. This means that **DT** interferes with the topo IIα catalytic cycle [35]. However, the compound did not generate linear DNA [35], which is associated with the stabilization of topo II-DNA cleavage complex typical of topo II poisons [37].

The link between the inhibition of topo IIα and the apoptotic activity of **DT** is controversial. **DT** increased the apoptotic fraction of K562 cells at concentrations of 2.5, 5.0, and 10 μg/mL. Moreover, the compound at 0.5 and 1.0 μg/mL activated caspase-3 (Casp-3) and cleaved poly (ADP-ribose) polymerase (PARP), while at 5 μg/mL it decreased Casp-3 activity and PARP cleavage. **DT** also induced the phosphorylation of various DNA damage-related proteins, including H2A histone family member X (H2A.X), ataxia telangiectasia mutated (ATM), breast cancer gene (BRCA), and ataxia-telangiectasia rad3-related (ATR) in the same concentration-dependent manner. Additionally, while 2.5 μg/mL of **DT** increased intracellular reactive oxygen species (ROS) levels in a time-dependent manner (0–60 min), at 5 μg/mL, ROS levels rose up to 30 min and then gradually decreased time-dependently [35]. This could possibly explain the lower activation of Casp-3 and the lower phosphorylation of DNA damage-related proteins in cells treated with **DT** 5 μg/mL. At the same time, the pre-treatment of cells with the ROS scavenger N-acetyl cysteine (NAC) inhibited the apoptotic activity and the protein expression of phosphorylated H2A.X (γ-H2A.X) induced by **DT** at 5 μg/mL [35]. This result points out that, although inhibition of topo IIα is associated with the activation of DNA damage-related proteins, overproduction of ROS also contributes to increase DNA damage and seems to be the major pro-apoptotic trigger. ROS-induced apoptosis by **DT** has been found to involve the IKK (IκB kinases)/NFκB (nuclear factor kappa B) and PI3K (phosphatidylinositol 3-kinase)/Akt signaling pathways, as demonstrated by the reduced expression of IKK/NFκB-related proteins and the increased phosphorylation of Akt [35]. Given that the continuous activation of IKK/NF-κB pathway promotes tumorigenesis [38], its inhibition by **DT** could be considered an additional mechanism of its antitumor effect. However, Akt activation is associated with tumor aggressiveness and drug resistance [39]. Hence, further investigation should be carried out to clearly understand the effects of **DT** resulting from the activation of Akt.

Regarding **apl-1**, Shih and colleagues explored its antitumor activity on leukemic and prostatic cancer cell lines, focusing also on its ability to inhibit topo II. **Apl-1** was highly cytotoxic (Table 1) and induced apoptosis through the dysregulation of the oxidative balance, as demonstrated by the excess of ROS and NOX (active nicotinamide adenine dinucleotide phosphate oxidase) production [36]. In addition, **apl-1** reduced the activity of the PI3K/Akt/mTOR (mammalian target of rapamycin) pathway, a mechanism associated with an antitumor activity [40]. Moreover, **apl-1** inhibited the relaxation of supercoiled DNA, showing an IC_50_ (concentration that inhibited the 50% of DNA relaxation) value of 1.37 μM (Table 1). As **DT**, **apl-1** did not generate linear DNA [36], meaning that it could not stabilize the DNA cleavage complex. A further study determined that **apl-1**, despite increasing phosphorylation of H2A.X, did not produce DNA single strand breaks (SSBs) and DSBs, and did not increase the number of nuclear γ-H2A.X foci [41]. All these findings show that **apl-1**, in contrast to its oxidized derivative, acts as a topo IIα catalytic inhibitor, without inducing DNA damage.

**Apl-1** inhibited the protein expression of heat shock protein 90 (Hsp90) in PC-3 and Du145 prostate cancer cells, making it a dual target inhibitor [36]. Hsp90 chaperon ensures the stability, integrity, shape, and function of critical oncogenic proteins (also called Hsp90 client proteins), which play critical roles in signal transduction, cell proliferation and survival, cell-cycle progression and apoptosis, as well as invasion, tumor angiogenesis, and metastasis [42]. Other marine topo II inhibitors, in addition to **apl-1**, possess this dual inhibitory activity of topo II and Hsp90, as discussed in the next sections. This is probably due to the similar ATPase domain structures of topo II and Hsp90 [43]. Other studies found that **apl-1** inhibited the Wnt/β-catenin pathway through the proteasomal degradation of β-catenin [44] and the epidermal growth factor (EGF)-dependent proliferation of breast cancer cells (MCF-7 and ZR-75-1), probably by blocking the phosphorylation of EGF receptor [45]. Moving toward the later stages of the carcinogenic process, **apl-1** showed antimetastatic and antiangiogenic effects: in PC-3 and Du145 cells, it inhibited cell migration and colony formation, and suppressed the EMT process induced by the transforming growth factor-β1 (TGF-β1) [36].

Overall, **apl-1** exerted a marked antitumor activity in different tumor cell models and modulated multiple targets. Despite this, conflicting results are reported regarding its selective activity toward cancer cells. In normal rat macrophage cells (NR8383) and normal human skin cells (CCD966SK), the IC_50_, calculated for its cytotoxic effects, was almost 4− and 17−fold higher, respectively, than the average IC_50_ calculated for tumor cells (0.39 μM) [36]. However, **apl-1** induced apoptosis and blocked cell-cycle progression indiscriminately in leukemia (THP-1 and NOMO-1) cells and in bovine aortic endothelial cells [41]. Thus, the toxicological profile of **apl-1** needs more in-depth studies.

### 2.3. Makaluvamines

Another type of alkaloids produced by sponges are pyrroloiminoquinones, which include makaluvamines and batzellines.

**Figure 4:**
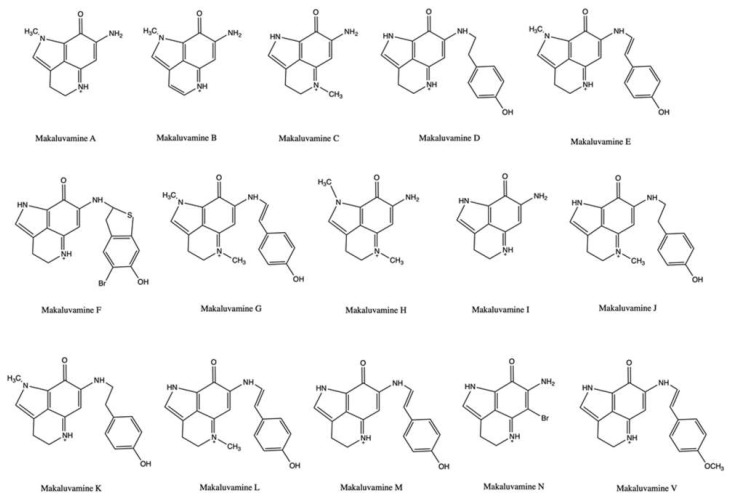
Chemical structure of makaluvamines A-V. Makaluvamine A (CAS number: 146555-78-4), makaluvamine B, C (CAS number: not available), makaluvamine D (CAS number: 146555-81-9), makaluvamine E (CAS number: 146555-82-0), makaluvamine F (CAS number: 146555-83-1), makaluvamine G (CAS number: 152273-69-3), makaluvamine H (CAS number 174232-34-9), makaluvamine I (CAS number: 138087-43-1), makaluvamine J (CAS number:174232-35-0), makaluvamine K (CAS number: 174232-36-1), makaluvamine L (CAS number: 174232-37-2), makaluvamine M (CAS number: 174232-41-8), makaluvamine N (CAS number: 187964-02-9), makaluvamine V (CAS number: 227103-87-9).

Makaluvamines (Figure 4) were isolated from sponges mainly belonging to the *Zyzza* genus. In the 1990s, these compounds were the subject of intensive studies to evaluate their antitumor activity. All makaluvamines (A-V) exhibited a marked cytotoxic activity. [46,47,48]. In addition, makaluvamine A and C reduced the tumor mass of human ovarian carcinoma OVCAR3-xenograft in Balb/c nu/nu athymic mice (Table 1) in vivo [49]. 

Regarding the ability of makaluvamines to inhibit topo II, the results are somewhat ambiguous: makaluvamine G did not inhibit topoisomerase II; for the other makaluvamines, there are conflicting data on whether they act as topo II catalytic inhibitors or poisons. Makaluvamine N inhibited more than 90% of the relaxation of supercoiled pBR322 DNA at 5.0 μg/mL [46,49], while makaluvamines A-F modulated topo II-mediated decatenation of kinetoplast DNA (kDNA) differently [49,50]. Overall, makaluvamine B was inactive, while makaluvamine A and F were the most effective, exhibiting IC_90_ (concentration that inhibits 90% of kDNA decatenation) values of 41 μM and 25 μM, respectively [49]. Later, Matsumoto et al. demonstrated that different makaluvamines promoted the formation of cleavable complex. Makaluvamine C, D, and E (33–466 μM) cleaved radiolabeled pUC 19 DNA in the presence of human topo II in a concentration-dependent manner, although they showed fewer and weaker cleavage sites than ETO and mitoxantrone. In addition, when also testing other makaluvamines at 91 mM using a cell-free cleavage assay with radiolabeled rf M13 mp 19 plasmid DNA, they found that makaluvamine I and H were the most efficient in inducing topo II-mediated cleavage of plasmid DNA, showing a 61% and 33% of cleavage, respectively, compared to the 100% of ETO, at the same tested concentration (Table 1). In both assays, makaluvamine D and E exhibited a comparable behavior, i.e., a weak and marked formation of cleavable complex, respectively, whereas makaluvamine C was more efficient in cleaving plasmid DNA than radiolabeled pUC 19 DNA [51]. Overall, this latter study points out that makaluvamines may act as topo II poisons. In support of this hypothesis, there are various data. Firstly, makaluvamine A intercalated into DNA and induced DNA DSBs in the neutral filter elution assay, which measures the formation of protein-linked DNA DSBs, compatible with the generation of DNA cleavable complex. The effect was comparable to that of the known DNA intercalating topo II poison m-AMSA [49]. Similar findings were reported for makaluvamine C [50]. Secondly, the most active makaluvamines (A and F) were much more cytotoxic in CHO xrs-6 cells compared to CHO BR1 cells (DSBs repair-competent): they exhibited a hypersensitive factor (HF, i.e., the ratio of IC_50_ on xrs-6 to that on BR1 cells) equal to 9 (for makaluvamine A) and 6 (for makaluvamine F), and thus equal to or higher than that of m-AMSA (HF = 6) [49]. Similarly, makaluvamine I showed a 5-fold lower IC_50_ in xrs-6 cells (0.4 μM) compared to AA8 DNA repair-competent cells (2 μM) [51]. This evidence shows a typical behavior of DNA intercalating topo II poisons. Overall, it is very likely that some makaluvamines have the formation of cleavable complexes as their predominant mechanism and thus act as a poison. However, the lack of extensive studies does not allow to clearly identify the mechanism of topo II inhibition of the different compounds. In addition, further experiments on their activity on in vitro or in vivo models are needed to identify their potential use as anticancer agents.

Recently, different makaluvamine analogs as well as a hybrid derived from makaluvamine A and ellipticine have been found to inhibit the catalytic activity of topo II and block DNA relaxation [52,53]. However, the hybrid derivative was equally cytotoxic on both prostate cancer cells and normal fibroblasts, thus demonstrating a non-selective activity toward tumor cells [53].

### 2.4. Batzellines

**Figure 5:**
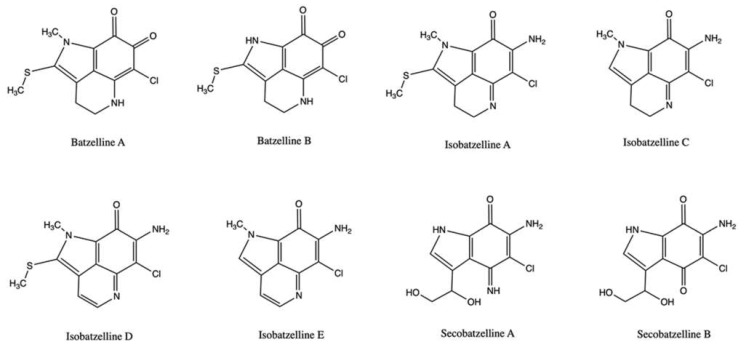
Chemical structure of batzellines. Batzelline A (CAS number: 123064-89-1), batzelline B (CAS number: 123064-90-4), isobatzelline A (CAS number: 133401-01-1), isobatzelline C (CAS number: 133401-03-3), isobatzelline D (CAS number: 133401-04-4), isobatzelline E (CAS number: 437980-21-7), secobatzelline A (CAS number: 247590-59-6), secobatzelline B (CAS number: 247590-60-9).

Batzellines are a group of alkaloids isolated from the marine sponge *Batzella* sp. (Figure 5), structurally linked to other marine substances such as makaluvamines and discorhabdins. 

Among them, isobatzelline A, isobatzelline C, isobatzelline D, and secobatzelline A were highly cytotoxic on a panel of pancreatic cancer cell lines (Table 1). Surprisingly, cytotoxic activity was found to be inversely proportional to the inhibition of topo II-mediated DNA decatenation [54]. Isobatzelline E and batzelline B, which are not among the most cytotoxic, inhibited 95% and the 63%, respectively, of DNA decatenation at 25 μg/mL; at the same concentration, isobatzellines A, C, and D, which are the most cytotoxic, inhibited 36%, 27%, and 26% of topo II-mediated DNA decatenation, respectively. These latter significantly intercalated into DNA, while the most potent topo II inhibitor isobatzelline E was the less potent DNA-intercalating compound [54]. This different behavior seems to influence the mechanism by which batzellines interfere with cell-cycle progression in a different way. In fact, only the most potent topo II inhibitor isobatzelline E blocked cells in the G2 phase of the cell cycle, whereas all the others, characterized by a less pronounced inhibitory activity on topo II and a greater ability to intercalate into DNA, blocked cell-cycle progression in the S phase [54]. Overall, these results indicate that batzellines cytotoxicity relies upon both topo II inhibition and DNA-intercalation, and that the more batzellines intercalate into the DNA, the greater the cytotoxicity of the specific compound [54]. Bearing in mind the close similarity with makaluvamines and, especially, the marked ability of isobatzellins A, C, D to intercalate with DNA, more in-depth studies should be carried out to assess whether batzellines induce DNA damage and act as topo II poisons by promoting the formation of DNA cleavable complex. 

### 2.5. Hippospongic Acid A

Hippospongic acid A (HA-A) is a triterpene isolated from the marine sponge *Hippospongia* sp.

**Figure 6:**
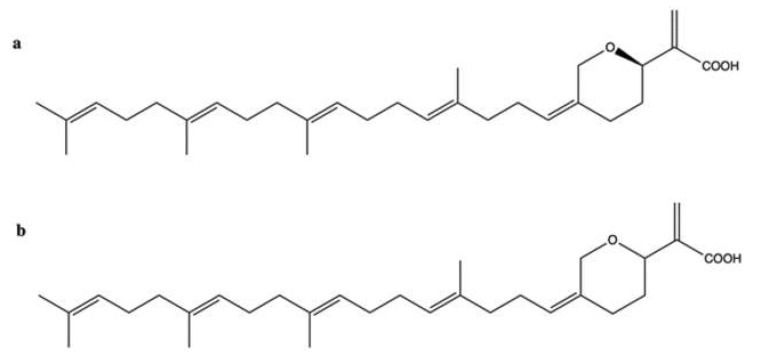
Chemical structure of (*R*)-HA-A ((**a**) CAS number: not available) and (±)-HA-A ((**b**) CAS number: 183381-06-8).

Both the natural enantiomer (*R*)-HA-A (Figure 6a) and the racemate (±)-HA-A (Figure 6b), which consists of the natural stereoisomer [(*R*)-HA-A] and the unnatural one [(*S*)-HA-A], dose-dependently inhibited both human and yeast topo II relaxation activity, showing an IC_50_ value of 15 μM. Inhibition of topo I has also been observed, although with a higher IC_50_ value (25 μM), together with the inhibition of DNA polymerases within 2-fold higher IC_50_ values [55]. (*R*)-HA-A and (±)-HA-A at 10 μM blocked cell-cycle progression in both G1 and G2/M phases, and induced apoptosis in NUGC-3 human gastric cancer cells. The G1-phase arrest was probably due to the inhibition of DNA polymerases, while the G2/M-phase block was mainly due to the inhibition of topoisomerases [55]. Based on these results, it seems likely that several mechanisms, namely inhibition of topo I, topo II, and DNA polymerases, are involved in the compound’s antitumor activity rather than the exclusive inhibition of topo II.

### 2.6. 10-Acetylirciformonin B

**Figure 7:**
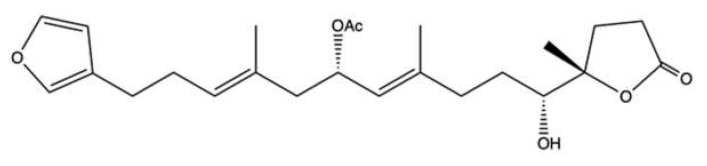
Chemical structure of 10-Acetylirciformonin B (CAS number: 1334233-11-2).

10-Acetylirciformonin B (**10AB**) (Figure 7) is a furanoterpenoid derivative isolated with other terpenoid-derived metabolites from the marine sponge *Ircinia* sp. [56]. 

Among all the isolated compounds, **10AB** was the most cytotoxic (Table 1). Interestingly, it seems to exert a selective cytotoxic effect for cancer cells: in HL-60 cells, **10AB** at 6.0 μM induced 80% apoptosis; in rat alveolar NR8383macrophages, it suppressed cell viability by 18.3% [57]. A previous study reported that in HL-60 cells **10AB** induced Casp-dependent apoptosis and promoted the formation of DNA DSBs, accompanied by the phosphorylation of H2A.X and checkpoint kinase 2 (Chk2), two markers of nuclear DNA damage [58]. A more recent study showed that **10AB**-induced DNA damage may be related to its ability to inhibit topo IIα catalytic activity: **10AB** (1.5, 3.0, 6.0, and 12.0 μM) inhibited DNA relaxation without producing linear DNA (like the topo IIα poison ETO), and at 3 μM decreased the protein expression of topo IIα in HL-60 cells. All these findings indicate that **10AB** could act as a DNA damaging agent and compromise the topo IIα catalytic cycle, leading to apoptotic cell death [57]. In this regard, in HL-60 cells **10AB** (1.5, 3.0, and 6.0 μM) disrupted MMP (mitochondrial membrane potential) and reduced the protein expression of anti-apoptotic proteins (Bcl-2 and Bcl-X) as well as of other proteins involved in the apoptotic process, as X-linked inhibitor of apoptosis protein (XIAP) and survivin. **10AB** also generated ROS, activated the mitogen-activated protein kinases (MAPK)/extracellular signal-regulated kinase (ERK) pathway, and inhibited the PI3K/PTEN/Akt/mTOR signaling pathway [57]. Akt transcriptionally regulates the expression of hexokinase II (HK-II) [59]. HKs are enzymes that catalyze the phosphorylation of glucose, i.e., the first step of glycolysis, and are upregulated in many tumors characterized by a high glycolytic activity. Moreover, HK-II has a pro-survival activity and protects mitochondria against mitochondrial apoptotic cell death by interfering with anti- and pro-apoptotic proteins and decreasing ROS generation [59]. Thus, downregulation of HK allows the shift of cancer cells’ metabolism to oxidative phosphorylation and increases ROS levels, which leads to cell death. The demonstrated ability of **10AB** to downregulate p-Akt protein expression may lead to the downregulation of HK-II. This means that **10AB**-induced apoptosis seems to be mediated by topo IIα inhibition and oxidative stress, as well as the perturbation of metabolic and cell survival pathways.

### 2.7. Manoalide-Like Sesterterpenoids

**Figure 8:**
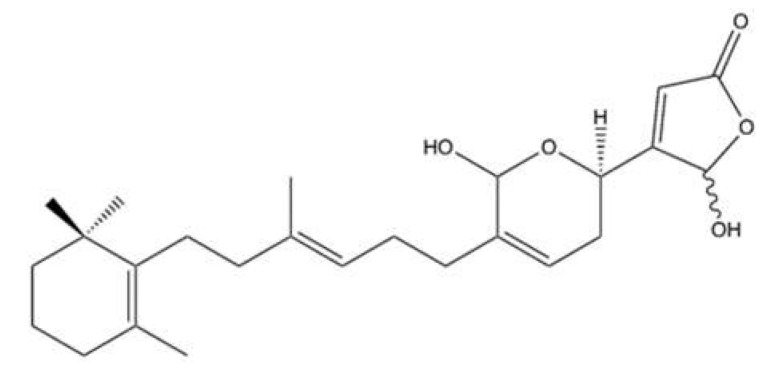
Chemical structure of manoalide 25-acetals (CAS number: not available).

In 1994, Kobayashi et al. isolated four sesterterpenes from the sponge *Hyrtios erecta* [60]. Among them, manoalide 25-acetals (Figure 8) inhibited the DNA-unknotting activity of calf thymus topo II, showing an IC_50_ value of about 25 μM. In addition, it exhibited antitumor activity on CDF_1_ mice inoculated whit P388 leukemia cells, with a T/C% score (the ratio between the tumor volume in the treated group and in the untreated control group) of 150% at 1 mg/kg (Table 1) [60]. 

**Figure 9:**
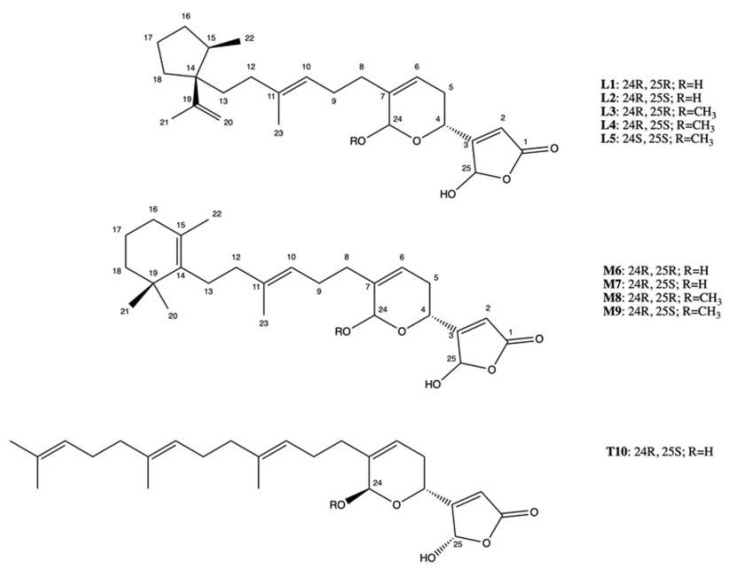
Chemical structure of the manoalide-like sesterterpenoids. M1-M5 (CAS numbers not available), M6 (CAS number: 2328074-79-7), M7-M9 (CAS numbers not available), T10 (CAS number not available).

More recently, 10 manoalide-like sesterterpenoids (Figure 9) were isolated from *Luffariella* sp. sponge: 24*R*,25*R*-luffariellin A (**L1**), 24*R*,25*S*-luffariellin A (**L2**), 24*R*-*O*-Methyl-25*R*-luffariellin A (**L3**), 24*R*-*O*-Methyl-25*S*-luffariellin A (**L4**), 24*S*-*O*-Methyl-25*S*-luffariellin A (**L5**), 24*R*,25*R*-manoalide (**M6**), 24*R*,25*S*-manoalide (**M7**), 24*R*-*O*-Methyl-25*R*-manoalide (**M8**), 24*R*-*O*-Methyl-25*S*-manoalide (**M9**), and 24*R*,25*S*-thorectolide (**T10**) [61].

All the derivates were tested on multiple leukemia cell lines (Table 1). The compounds **L2**, **L4**, **M7**, and **M9**, bearing a 24R, 25S configuration, were the most effective, thus assuming that the cytotoxic activity was configuration-dependent [61]. The administration of **M7** to immunodeficient athymic mice (1 μg/kg every day for 33 days) reduced the tumor growth of Molt-4 xenograft by about 66%, without affecting body weight [61].

**M7** has been shown to act as a catalytic inhibitor of topo IIα. Moreover, it inhibited DNA relaxation with an IC_50_ value of 1.18 μM and promoted the formation of supercoiled DNA products in the presence of topo IIα [61]. Compared to manoalide 25-acetals, the inhibitory activity of **M7** toward topo II was greatly higher, although purified topo II from two different organisms were used: human for **M7** [61] and calf thymus for manoalide 25-acetals [60]. The topo IIα catalytic inhibitor activity was associated with DNA damage, as demonstrated by its ability to promote the phosphorylation of ATM, Chk2, and H2A.X and to induce DNA DSBs at 0.75 μM in Molt-4 cells. **M7**-induced DNA damage has been found to activate apoptotic cell death, as indicated by and the activation of Casp-3, -8, and -9, the disruption of MMP, and the cleavage of PARP [61]. 

### 2.8. Heteronemin

**Figure 10:**
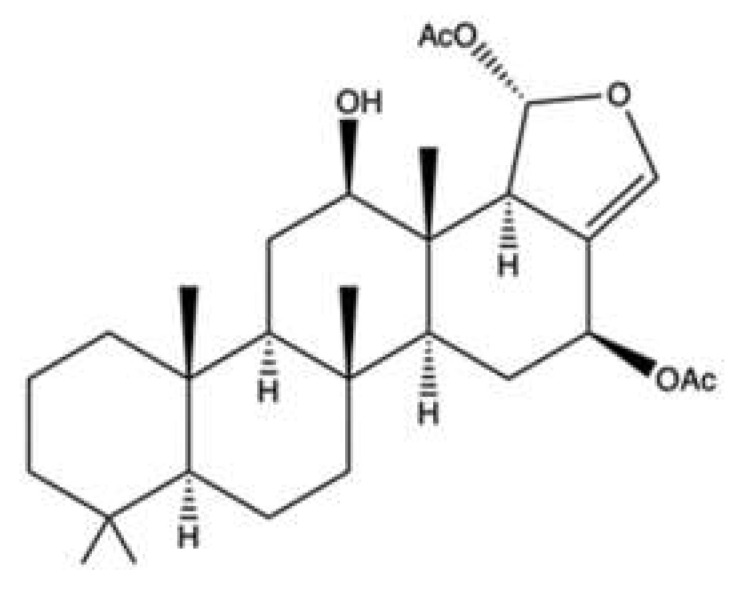
Chemical structure of heteronemin (CAS number: 62008-04-2).

Another marine sesterterpenoid-type product, heteronemin (Figure 10), was separated from the *Hippospongia* sp. sponge [62].

Heteronemin was able to induce apoptosis as well as inhibit the proliferation of different cancer cell lines [63,64]. Interestingly, in hepatocellular carcinoma HA22T and HA59T cells, heteronemin induced both apoptosis and ferroptosis [65], a non-apoptotic programmed cell death mechanism characterized by the iron-dependent accumulation of lipid ROS [66]. Due to the well-known occurrence of multi-drug resistance caused by the deregulation of apoptosis [67], the evidence that heteronemin is a ferroptosis inducer is very interesting.

Deepening the molecular mechanisms involved in heteronemin’s cytotoxicity in prostate cancer cells, Lee et al. found that it induced both autophagy and apoptosis [62]. Autophagy promotes either cell survival or cell death in a context- and cell-dependent manner [68]. Autophagy induced by heteronemin seems to possess a cytoprotective effect rather than a pro-apoptotic one [62]. Indeed, heteronemin (1.28 and 2.56 μM) activated LC3-B II (LC3-phosphatidylethanolamine conjugate), a marker of autophagy, but at 5.12 μM, when apoptosis was markedly induced, autophagy was blocked. Moreover, the pre-treatment with two autophagy inhibitors (3-methyladenine and chloroquine) raised the percentage of LNCaP apoptotic cells [62]. Similarly, in A498 renal carcinoma cells, the inhibition of autophagy increased the pro-apoptotic activity of heteronemin [69].

The marine sesterterpene completely inhibited DNA relaxation in the cell-free DNA cleavage assay and reduced topo IIα protein expression in LNCaP cells, which resulted in the block of the total catalytic activity of the enzyme. Heteronemin did not produce linear DNA, thus assuming its inability to stabilize DNA-topo II cleavable complex [62]. 

Mechanisms other than the inhibition of topo II are possibly involved in the antitumor activity of heteronemin. 

Heteronemin suppressed the expression of Hsp90 and that of its client proteins, thus being able to modulate the expression of oncogenic proteins and transcription factors involved in tumorigenesis [62]. Moreover, it blocked NF-κB activation via proteasome inhibition in K562 cells [70] and the activation of ERK1/2 and STAT3 in breast cancer cells [63,64]. In LnCaP cells, heteronemin (1.28–5.12 μM) disrupted MMP, fostering mitochondrial dysfunction. Due to the overproduction of ROS and Ca^2+^ release, heteronemin promoted oxidative and endoplasmic reticulum (ER) stress, therefore triggering the unfolded protein response (UPR) signaling network to re-establish ER homeostasis [62]. Oxidative and ER stress results from the activation of protein tyrosine phosphatases (PTPs) [62]. PTPs modulate the levels of cellular protein tyrosine phosphorylation and control cell growth, differentiation, survival, and death. PTPs exert both tumor-suppressive and oncogenic functions in a context-dependent manner [71]. Pre-treatment of LnCaP with a PTP inhibitor reduced heteronemin-induced ROS generation and ER stress, thus demonstrating that in this experimental setting, PTPs exhibits a tumor-suppressive mechanism and participates in the antitumor activity of heteronemin [62]. 

Oxidative stress was also involved in the heteronemin-induced anticancer effects in Molt-4 cells. In this cell line, it enhanced γ-H2A.X protein expression, probably due to apoptosis rather than DNA damage occurrence. Indeed, although γ-H2A.X is the most sensitive biomarker of DNA damage, its measure by ELISA and/or immunoblotting allows to evaluate the total H2A.X protein levels in a sample, but apoptotic cells with pan-nuclear H2A.X expression cannot be differentiated from surviving cells, which may alter H2A.X quantification. In contrast, the fluorescent microscopic quantification of foci is the most sensitive approach and can distinguish between pan-nuclear staining and foci formation [72]. The increased γ-H2A.X protein expression induced by heteronemin in Molt-4 cells was demonstrated by using Western Blot, as for all the other sponge-derived topo II inhibitors, and, unlike other studies, the expression of other DNA damage-related proteins was not evaluated. Thus, it is not clear whether heteronemin induces DNA damage in this experimental model.

In vivo, heteronemin inhibited the growth of Molt-4 and LnCaP xenograft in Balb/c nude mice and in immunodeficient athymic mice, respectively, treated with 0.31 μg/g (three times a week for 24 days) and 1 mg/kg (every day for 29 days) of heteronemin [62,73].

### 2.9. Scalarane Sesterterpenoids

**Figure 11:**
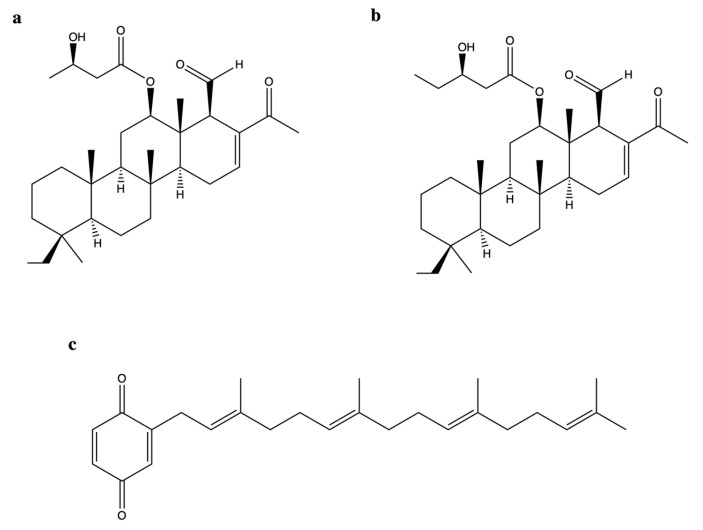
Chemical structure of 12*β*-(3′*β*-hydroxybutanoyloxy)-20,24-dimethyl-24-oxo-scalara-16-en-25-al (**a**), 12*β*-(3′*β*-hydroxypentanoyloxy)-20,24-dimethyl-24-oxo-scalara-16-en-25-al (**b**), and 2-tetraprenil-1,4-benzoquinone ((**c**) CAS numbers not available).

**Figure 12:**
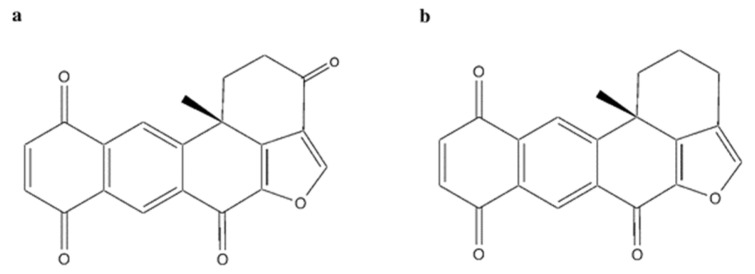
Chemical structure of halenaquinone ((**a**), CAS number: 86690-14-4) and xestoquinone ((**b**), CAS number: 97743-96-9).

A dual inhibitory effect on topo II and Hsp90 was also reported for two new scalarane sesterterpenoids (SS) [12*β*-(3′*β*-hydroxybutanoyloxy)-20,24-dimethyl-24-oxo-scalara-16-en-25-al (**SS1**, Figure 11a) and 12*β*-(3′*β*-hydroxypentanoyloxy)-20,24-dimethyl-24-oxo-scalara-16-en-25-al (**SS2**, Figure 11b)], and a tetraprenyltoluquinol-related metabolite (2-tetraprenil-1,4-benzoquinone, **TPL**, Figure 11c), all isolated from the sponge *Carteriospongia* sp. [74]. 

**SS1**, **SS2**, and **TPL** were cytotoxic on many tumor cell lines [74] (Table 1). All three compounds inhibited DNA relaxation, reaching almost 100% inhibition at the highest tested concentration (20 μg/mL). There was no information regarding the production of linear DNA [74]. Topo II inhibition was associated with DNA damage: **SS1** (0.0625–0.25 μg/mL) increased the protein expression of γ-H2A.X and, at 0.0625 μg/mL; it also induced DNA DSBs in Molt-4 cells [74]. Although **SS2** enhanced γ-H2A.X protein expression, it is difficult to associate this event exclusively with DNA damage since neither other marker of DNA damage nor the formation of DSBs have been evaluated. **SS1**, like heteronemin [62], promoted ROS generation and ER stress and induced mitochondrial apoptosis [74]. In addition, **SS1** shared with heteronemin the ability to inhibit Hsp90 protein expression and that of its client proteins [74]. Although Lai and colleagues investigated **SS1** more deeply than **TPL**, the latter was also tested in a Molt-4 cells xenograft animal model, showing that its daily administration (1.14 μg/g) for 33 days inhibited almost 50% of xenograft tumor growth in male immunodeficient athymic mice [74]. Authors justified their choice to only test **TPL** in vivo by the small amount they were able to isolate for the other two compounds. However, considering the marked antitumor activity of **SS1**, a possible in vivo study of this compound should be considered as well.

### 2.10. Polycyclic Quinone-Type Metabolites 

The two polycyclic quinone-type compounds halenaquinone [75] (Figure 12a) and xestoquinone [76] (Figure 12b), which differ for a carbonyl group, were isolated from the sponge *Petrosia* sp. 

Halenaquinone and xestoquinone exhibited a comparable cytotoxic activity [75,76]. In vivo, the administration of halenaquinone (1 μg/g for 30 days) and xestoquinone (1 μg/g for 50 days) suppressed the growth of Molt-4 xenograft in immunodeficient athymic mice, without affecting body weight (Table 1) [75,76].

Both compounds strongly inhibited either the topo II-catalyzed DNA relaxation and the protein expression of topo IIα in Molt-4 [75,76] and K562 cells [76]. For DNA relaxation, xestoquinone showed an IC_50_ value of 0.094 μM [76], and halenaquinone showed an IC_50_ about 5.5-fold lower (0.017 μM) [75]. These results indicate that they act as potent catalytic inhibitors of topo II. However, they did not form DNA-topo II cleavage complex, since no linear DNA was observed in the cell-free DNA relaxation assay [75,76]. Additionally, molecular docking studies reported that xestoquinone was capable of binding topo II with a docking score of −26.9, although a similar or even a lower value was observed for topo I (−24.0) and Hsp90 (−15.5) [76]. These results demonstrate that the compound can bind to multiple targets. Xestoquinone (7.84 μM) treatment of Molt-4 cells markedly increased the expression of multiple DNA damage markers (p-Chk1, p-Chk2, and γ-H2A.X), pointing out that its topo II catalytic activity inhibition induced DNA damage [76]. No markers of DNA damage were evaluated for the congener halenaquinone. Nonetheless, given the close similarities in the antitumor mechanisms of both compounds, it cannot be excluded that congener halenaquinone was a topo II catalytic inhibitor. In fact, both compounds have been shown to inhibit the activity of histone deacetylase (HDAC) in vitro [75,76] and in a Molt-4 xenograft mouse in vivo model [76]. This is not so surprising, as several studies report that topo II and HDAC mutually modulate their activity [43]. In addition to this, ROS overproduction [75,76], induction of ER stress, and binding to protein Hsp90 [76] recorded for both compounds led to apoptosis. Notably, the two polycyclic quinone-type metabolites promoted both apoptotic pathways as the disruption of MMP, decrease in anti-apoptotic proteins (Bcl-2, Bcl-X, Bid), increase in pro-apoptotic ones (Bax, Bak) (all markers of intrinsic apoptosis), and activation of Casp-8 and -9 (markers of extrinsic apoptosis) were observed in Molt-4 and K562 cells [75,76]. 

**Figure 13:**
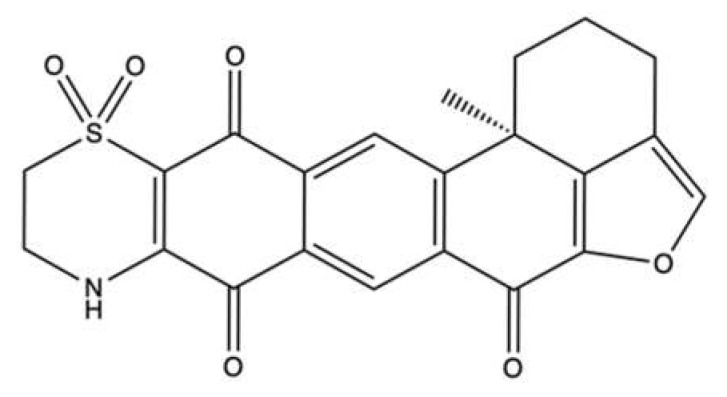
Chemical structure of adociaquinone B (CAS number: 113831-00-8).

Alongside halenaquinone and xestoquinone, other polycyclic quinone-type metabolites were isolated from the sponge *Xestospongia* sp. [77]. All studied compounds inhibited topo II (Table 1). Among those, adociaquinone B (Figure 13) was the most potent with an IC_90_ (the concentration inducing the 90% of inhibition) < 11 μM and 78 μM for DNA decatenation and relaxation, respectively. In contrast to xestoquinone and halenaquinone, adociaquinone B was a non-intercalating DNA topo II poison. In fact, it strongly promoted the formation of the enzyme-DNA cleavable complex to the same extent as mitoxantrone, a known topo II poison [78]. However, in contrast to mitoxantrone, adociaquinone B did not intercalate into DNA since it was not able to displace ethidium bromide from calf thymus DNA [77]. Secoadociaquinone A and B, two other *Xestospongia* sp. metabolites, inhibited topo II activity in the cell-free DNA decatenation assay without exhibiting cytotoxicity since they were unable to permeate cell membranes. Thus, it is not sufficient to test the inhibitory activity of topo II only on cell-free systems, as very often the physicochemical properties of the tested compounds prevent their entry into cells and consequently a possible interaction with intracellular targets, such as topo II [77].

**Table 1:** Sponge-derived inhibitors of topo II.

Compound	Sponge Species (Sp.)	Topo II Inhibitory Activity			Antitumor Effect(s)			Ref.
		Assay	IC_50_/IC_90_^a^ or Range of Concentrations Tested	Outcomes	Experimental Model	Cytotoxic Activity (IC_50_^b^)	Other Antitumor Mechanism(s)	
(1′R,5′S,6′S)-2-(3′,5′-dibromo-1′,6′-dihydroxy-4′-oxocyclohex-2′-enyl) acetonitrile	*Pseudoceratina* sp.	Cell-free DNA cleavage assay using an enzyme-mediated negatively supercoiled pHOT1 plasmid DNA and human topo II	2.5–10 μg/mL	↓ DNA relaxation in presence of topo IIα	K562	IC_50_ (72 h): 1.4 μg/mL	Apoptosis (↑ cleaved PARP, ↑ cleaved caspase-3, ↑ XIAP protein expression) DNA damage (↑ p-ATM, ↑ p-ATR, ↑ γH2A.X protein expression, ↑ p-BRCA, ↑ p-Chk2 protein expression) Oxidative stress (↑ ROS) ↓ IKK/NFκB pathway ↑ PI3K/Akt pathway	[35]
					HeLa	IC_50_ (72 h): 4.8 μg/mL		
					MCF-7	IC_50_ (72 h):1.9 μg/mL		
					MDA-MB-231	IC_50_ (72 h): 5.5 μg/mL		
2-tetraprenil-1,4-benzoquinone	*Carteriospongia* sp.	Cell-free DNA cleavage assay using an enzyme-mediated negatively supercoiled pHOT1 plasmid DNA and human topo II	IC_50_: 0.43 μg/mL	↓ DNA relaxation and formation of supercoiled DNA products in presence of topo IIα	Molt-4	IC_50_ (72 h): 0.34 μg/mL	Apoptosis (↓ MMP)	[74]
					K562	IC_50_^b^ (72 h): 0.70 μg/mL		
					HL-60	IC_50_ (72 h): 0.42 μg/mL		
					U937	IC_50_ (72 h): 0.65 μg/mL		
					Sup-T1	IC_50_ (72 h): 0.33 μg/mL		
					Ca9-22	IC_50_ (72 h): 0.97 μg/mL		
					Cal-27	IC_50_ (72 h): 0.51 μg/mL		
					LNCaP	IC_50_ (72 h): >20 μg/mL		
					DLD-1	IC_50_ (72 h): 15.41 μg/mL		
					T-47D	IC_50_ (72 h): 1.06 μg/mL		
					Immunodeficient athymic mice bearing Molt-4 xenograft		↓ Tumor growth (1.14 μg/g/day, for 33 days)	
10-acetylirciformonin B	*Ircinia* sp.	Cell-free DNA cleavage assay using an enzyme-mediated supercoiled pHOT1 plasmid DNA and human topo II	1.5–6.0 μM	↓ DNA relaxation and formation of supercoiled DNA products in the presence of topo IIα	HL-60	Not indicated	Apoptosis (↓ MMP; ↓ Bcl-2, ↓ Bcl-xL, ↓ XIAP, ↓ survinin, ↑ BAX, ↑ cleaved PARP protein expression, ↑ cyt c release) ↓ p-Akt, ↓ p-PTEN, ↓ Src, ↓ HKII, ↓ PKM 2, ↑ p-ERK, ↑ p-38, ↑ p-JNK, ↑ p-GSK 3β protein expression Oxidative stress (↑ ROS)	[57]
		Western Blotting on HL-60 cells	3.0 μM	↓ Topo IIα protein expression				
12*β*-(3′*β*-hydroxybutanoyloxy)-20,24-dimethyl-24-oxo-scalara- 16-en-25-al	*Carteriospongia* sp.	Cell-free DNA cleavage assay using an enzyme-mediated negatively supercoiled pHOT1 plasmid DNA and human topo II	IC_50_: 1.98 μg/mL	↓ DNA relaxation and formation of supercoiled DNA products in presence of topo IIα	K562	IC_50_ (72 h): 0.01 μg/mL		[74]
					Molt-4	IC_50_ (72 h): 0.01 μg/mL	Apoptosis (↓ MMP; ↑ cleaved caspase-8, ↑ cleaved caspase-9, ↑ cleaved PARP protein expression) ↑ DNA damage (↑ γH2AX protein expression, ↑ DNA DSBs) Oxidative stress (↑ ROS) ↑ ER stress (↑ Ca^2+^ release; ↑ IRE 1α, ↑ Bip, ↑ CHOP, ↑ Grp94, ↑ Hsp70, ↑ ATF6, ↓ PERK protein expression) ↓ Hsp90 (↓ Akt, ↓ p70S6k, ↓ NFκB, ↓ Raf-1, ↓ p-GSK3β, ↓ XIAP, ↓ MDM 2 ↓ Rb2, ↓ CDK4 ↓Cyclin D3, ↓ HIF 1 ↓ HSF1; ↑ Hsp70 protein expression)	
					HL-60	IC_50_ (72 h): 0.01 μg/mL		
					U937	IC_50_ (72 h): 0.01 μg/mL		
					Sup-T1	IC_50_ (72 h): 0.13 μg/mL		
					Ca9-22	IC_50_ (72 h): 0.10 μg/mL		
					Cal-27	IC_50_ (72 h): 0.56 μg/mL		
					LNCaP	IC_50_ (72 h): 13.87 μg/mL		
					DLD-1	IC_50_ (72 h): 2.33 μg/mL		
					T-47D	IC_50_ (72 h): 2.19 μg/mL		
12*β*-(3′*β*-hydroxypentanoyloxy)-20,24-dimethyl-24-oxo-scalara-16-en-25-al	*Carteriospongia* sp.	Cell-free DNA cleavage assay using an enzyme-mediated negatively supercoiled pHOT1 plasmid DNA and human topo II	IC_50_: 0.37 μg/mL	↓ DNA relaxation	K562	IC_50_ (72 h): 0.35 μg/mL		[74]
					Molt-4	IC_50_ (72 h): 0.30 μg/mL	Apoptosis (↓ MMP) DNA damage (↑ γH2AX protein expression)	
					HL-60	IC_50_ (72 h): 0.22 μg/mL		
					U937	IC_50_ (72 h): 0.61 μg/mL		
					Sup-T1	IC_50_ (72 h): 0.42 μg/mL		
					Ca9-22	IC_50_ (72 h): 1.48 μg/mL		
					Cal-27	IC_50_ (72 h): 3.17 μg/mL		
					LNCaP	IC_50_ (72 h): >20 μg/mL		
					DLD-1	IC_50_ (72 h): 1.71 μg/mL		
					T-47D	IC_50_ (72 h): 1.87 μg/mL		
14-methoxy-xestoquinone	*Xestopongia* sp.	Cell-free DNA relaxation assay with supercoiled pBR322 DNA plasmid and topo II of drosophila	IC_90_: 143 μM	↓ DNA relaxation	HCT-116	IC_50_ (18 + 72 h): 28 μM		[77]
					CHO xrs-6 ^c^	IC_50_ (18 + 72 h): 4.3 μM		
14-chloro-15-hydroxyxestoquinone	*Xestopongia* sp.	Cell-free decatenation reaction of kinetoplast DNA	IC_90_: 110 μM	↓ DNA decatenation	HCT-116	IC_50_ (18 + 72 h): 33 μM		[77]
		Cell-free DNA relaxation assay with supercoiled pBR322 DNA and topo II of drosophila	IC_90_: 135 μM	↓ DNA relaxation	CHO xrs-6 ^c^	IC_50_ (18 + 72 h): 27 μM		
15-methoxy-xestoquinone	*Xestopongia* sp.	Cell-free DNA relaxation assay with supercoiled pBR322 DNA and Topo II of drosophila	IC_90_: 143 μM	↓ DNA relaxation	HCT-116	IC_50_ (18 + 72 h): 28 μM		[77]
					CHO xrs-6 ^c^	IC_50_ (18 + 72 h): 4.3 μM		
15-chloro-14-hydroxyxestoquinone	*Xestopongia* sp.	Cell-free decatenation reaction of kinetoplast DNA	IC_90_: 110 μM	↓ DNA decatenation	HCT-116	IC_50_ (18 + 72 h): 33 μM		[77]
		Cell-free DNA relaxation assay with supercoiled pBR322 DNA and topo II of drosophila	IC_90_: 135 μM	↓ DNA relaxation	CHO xrs-6 ^c^	IC_50_ (18 + 72 h): 27 μM		
24*R*, 25*S*-Manoalide	*Luffariella* sp.	Cell-free DNA cleavage assay using an enzyme-mediated negatively supercoiled pHOT1 plasmid DNA and human topo II	IC_50:_ 1.18 μM	↓ DNA relaxation	Molt-4	IC_50_ (72 h): 0.82 μM	Apoptosis (↓ MMP; ↑ cleaved PARP, ↑ cleaved caspase-3, ↑ cleaved caspase-8, ↑ cleaved caspase-9 protein expression) DNA damage (↑ p-ATM, ↑ p-Chk2, ↑ γH2AX protein expression, ↑ DSBs) Oxidative stress (↑ ROS)	[61]
					K562	IC_50_ (72 h): 7.67 μM		
					Sup-T1	IC_50_ (72 h): 1.35 μM		
					U937	IC_50_ (72 h): 1.56 μM		
Adociaquinone A	*Xestopongia* sp.	Cell-free DNA relaxation assay with supercoiled pBR322 DNA and topo II of drosophila	IC_90_: 118 μM	↓ DNA relaxation	HCT-116	IC_50_ (18 + 72 h): 24 μM		[77]
					CHO xrs-6 ^c^	IC_50_ (18 + 72 h): 78 μM		
Adociaquinone B	*Xestopongia* sp.	Cell-free decatenation reaction of kinetoplast DNA	IC_90_: <11 μM	↓ DNA decatenation	HCT-116	IC_50_ (18 + 72 h): 21 μM		[77]
		Cell-free DNA relaxation assay with supercoiled pBR322 DNA and topo II of drosophila	IC_90_: 78 μM	↓ DNA relaxation	CHO xrs-6 ^c^	IC_50_ (18 + 72 h): 23 μM		
		KSDS assay	/	Formation of enzyme-DNA cleavable complex				
Aeroplysinin 1	*Pseudoceratina* sp.	Cell-free DNA cleavage assay using an enzyme-mediated negatively supercoiled pHOT1 plasmid DNA and human topo II	IC_50_: 1.37 μM	↓ DNA relaxation	Molt-4	IC_50_ (72 h): 0.12 μM	Apoptosis (↓ MMP; ↑ cleaved PARP, ↑ cleaved caspase-3, ↓ p-Akt, ↓ XIAP protein expression) Oxidative stress (↑ ROS; ↓ HIF-1 α, ↓ HO-1, ↑ catalase, ↑ MnSOD, ↓ NOX4, ↑ NOX2 protein expression) ↑ Hsp70 protein expression ↓ EGFR, ↓ p-EGFR, ↓ β-catenin protein expression	[36]
		Western blotting on Molt-4 cells	0.1–0.4 μM	↓ Topo IIα protein expression	K562	IC_50_ (72 h): 0.54 μM	Apoptosis (↓ MMP; ↑ cleaved PARP, ↑ cleaved caspase-3, ↓ p-Akt, ↓ XIAP protein expression) Oxidative stress (↑ ROS; ↓ HIF-1 α protein expression) ↓ β-catenin protein expression	
		Western blotting on PC-3 cells	0.8–3.2 μM	↓ Topo IIα protein expression	PC-3	IC_50_ (72 h): 0.58 μM	Apoptosis (↓ MMP; ↑ cleaved PARP, ↑ cleaved caspase-3, ↓ p-Akt, ↓ XIAP ↓ Bcl-2, ↓ p-mTOR protein expression) Oxidative stress (↑ ROS; ↓ HIF-1 α, ↓ HO-1, ↑ catalase, ↑ MnSOD, ↑ NOX4, ↓ NOX2 protein expression) ↓ Hsp90, ↑ Hsp70 protein expression ↓ Colony formation ↓ Cell migration ↓ EMT ↓ EGFR, ↓ p-EGFR, ↓ β-catenin protein expression	
					Du145	IC_50_ (72 h): 0.33 μM	Apoptosis (↓ MMP; ↑ cleaved PARP, ↑ cleaved caspase-3, ↓ p-Akt, ↓ XIAP ↓ Bcl-2, ↓ p-mTOR protein expression) Oxidative stress (↑ ROS; ↓ HIF-1 α, ↓ HO-1, ↑ catalase, ↑ MnSOD, ↓ NOX4, ↑ NOX2 protein expression) ↓ Hsp90 protein expression ↓ colony formation ↓ cell migration ↓ EMT	
					CCD966S ^d^	IC_50_ (72 h): 1.54 μM		
					NR8383 ^d^	IC_50_ (72 h): 6.77 μM		
Bastadin-14	*Psammaplysilla purpurea*	Not indicated	IC_50_: 2 μg/mL	↓ Topo IIα protein activity	A-549	IC_50_: 2 μg/mL		[79]
					HT-29			
					P388 ^e^			
					CV-1 ^d^	IC_50_: 2.5 μg/mL		
Batzelline A	*Batzella* sp.	Cell-free decatenation reaction of kinetoplast DNA	25 μg/mL (88.45 μM)	↓ DNA decatenation (58%)	Panc-1	IC_50_ (72 h): >17.68 μM		[54]
		Ethidium bromide displacement fluorescence assay	25 μg/mL (88.45 μM)	DNA intercalation (18%)	AsPC-1	IC_50_ (72 h): >17.68 μM	↓ Cell cycle in phase S	
					BxPC-3	IC_50_ (72 h): >17.68 μM		
					Mia PaCa2			
					Vero ^d^			
Batzelline B	*Batzella* sp.	Cell-free decatenation reaction of kinetoplast DNA	25 μg/mL (93.02 μM)	↓ DNA decatenation (63%)	Panc-1	IC_50_ (72 h): >18.61μM		[54]
		Ethidium bromide displacement fluorescence assay	25 μg/mL (93.02 μM)	DNA intercalation (21%)	AsPC-1	IC_50_ (72 h): >18.61μM	↓ Cell cycle in phase S	
					BxPC-3	IC_50_ (72 h): >18.61μM		
					Mia PaCa2			
					Vero ^d^			
Elenic acid	*Plakinastrella* sp.	Not indicated	IC_50_: 0.1 μg/mL	↓ Topo IIα activity	P388 ^e^	IC_50_ (time not indicated): 5 μg/mL		[80]
					A-549			
					MEL-28			
Halenaquinone	*Petrosia* sp.	Cell-free DNA cleavage assay using an enzyme-mediated negatively supercoiled pHOT1 plasmid DNA and human topo II	IC_50_: 0.0055 μg/mL (0.017 μM)	↓ DNA relaxation	Molt-4	IC_50_ (24 h): 0.61 μg/mL IC_50_ (72 h): 0.18 μg/mL	Apoptosis (↓ MMP; ↑ c-PARP, ↑ cleaved caspase-3, ↑ cleaved caspase-7, ↑ cleaved caspase-8, ↑ cleaved caspase-9, ↑ Bax, ↑ cyt c, ↓ Bcl-2, ↓ Bid protein expression) ↓ p-Akt, ↓ p-PTEN, ↓ p-GSK3β, ↓ p-PDK1 and ↓ HKII protein expression Oxidative stress (↑ ROS) Inhibition of HDAC activity (↑ acetyl-H3, ↑ acetyl-H3K18 protein expression)	[75]
		Western Blotting on Molt-4 cells	1.25 μg/mL	↓ Topo IIα protein expression	Immunodeficient athymic mice bearing Molt-4 xenograft		↓ Tumor growth, ↓ tumor weight, ↓ tumor volume (1μg/g/day for 30 days)	
					K562	IC_50_ (72 h): 0.48 μg/mL	Apoptosis (↑ cleaved PARP, ↑ cleaved caspase-3, ↑ cleaved caspase-7 protein expression)	
					MDA-MB-231	IC_50_ (72 h): 8 μg/mL		
					DLD-1	IC_50_ (72 h): 6.76 μg/mL		
Heteronemin	*Hippospongia* sp.	Cell-free DNA cleavage assay using an enzyme-mediated negatively supercoiled pHOT1 plasmid DNA and human topo II	2.56–40.9 μM	↓ DNA relaxation and formation of supercoiled DNA products in the presence of topo IIα	LNCaP	IC_50_ (24 h) 1.4 μM IC_50_ (48 h): 0.8 μM IC_50_ (72 h): 0.4 μM	Apoptosis (↓ MMP; ↑ cleaved PARP, ↑ cleaved caspase-3 protein expression) Oxidative stress (↑ ROS) ↑ ER stress (↑ Ca^2+^ release, ↑ IRE 1α, ↑ Bip, ↑ CHOP, ↑ Hsp70, ↓ ATF6, ↓ PERK protein expression) ↓ Hsp90, ↓ IRAK1, ↓ p-Akt, ↓ XIAP, ↓ Rb2, ↓ HDAC1, ↓ PCNA ↓ CDK4, ↓ p-STAT3, ↑ Hsp70 protein expression Autophagy (↑ LC3-II protein expression)	[62]
		Western blotting on LnCaP cells	0.64–2.56 μM	↓ Topo IIα protein expression	PC-3	IC_50_ (24 h): 2.7 μM	Apoptosis	
					Immunodeficient athymic mice bearing LNcaP xenograft	IC_50_ (24 h): 7 μM	↓ Tumor size, ↓ tumor growth (1 mg/Kg b.w.; for 29 days)	
Hippospongic acid A	*Hippospongia* sp.	Cell-free DNA relaxation assay using supercoiled pUC19 DNA plasmid and topo II	IC_50_: 15 μM	↓ DNA relaxation	NUGC-3	IC_50_ (time not indictaed): 9.5 μM	↓ Cell cycle in phases G1 and G2/M Apoptosis (↑ DNA fragmentation)	[55]
Isobatzelline A	*Batzella* sp.	Cell-free decatenation reaction of kinetoplast DNA	25 μg/mL (88.73 μM)	↓ DNA decatenation (36%)	Panc-1	IC_50_ (72 h): 9.37 μM		[54]
		Ethidium bromide displacement fluorescence assay	25 μg/mL (88.73 μM)	DNA intercalation (54%)	AsPC-1	IC_50_ (72 h): 1.74 μM	↓ Cell cycle in phase S	
					BxPC-3	IC_50_ (72 h): 2.39 μM		
					Mia PaCa2	IC_50_ (72 h): 4.34 μM		
					Vero^d^	IC_50_ (72 h): >17.75 μM		
Isobatzelline C	*Batzella* sp.	Cell-free decatenation reaction of kinetoplast DNA	25 μg/mL (106.38 μM)	↓ DNA decatenation (27%)	Panc-1	IC_50_ (72 h): 9.99 μM		[54]
		Ethidium bromide displacement fluorescence assay	25 μg/mL (106.38 μM)	DNA intercalation (56%)	AsPC-1	IC_50_ (72 h): 1.72 μM	↓ Cell cycle in phase S	
					BxPC-3	IC_50_ (72 h): 1.31 μM		
					Mia PaCa2	IC_50_ (72 h): 2.34 μM		
					Vero^d^	IC_50_ (72 h): >21.28 μM		
Isobatzelline D	*Batzella* sp.	Cell-free decatenation reaction of kinetoplast DNA	25 μg/mL (89.61 μM)	↓ DNA decatenation (26%)	Panc-1	IC_50_ (72 h): 5.72 μM		[54]
		Ethidium bromide displacement fluorescence assay	25 μg/mL (89.61 μM)	DNA intercalation (47%)	AsPC-1	IC_50_ (72 h): 1.48 μM	↓ cell cycle in phase S	
					BxPC-3	IC_50_ (72 h): 1.48 μM		
					Mia PaCa2	IC_50_ (72 h): 2.67 μM		
					Vero^d^	IC_50_ (72 h): 15.70 μM		
Isobatzelline E	*Batzella* sp.	Cell-free decatenation reaction of kinetoplast DNA	25 μg/mL (107.30 μM)	↓ DNA decatenation (95%)	Panc-1	IC_50_ (72 h): >21.46 μM		[54]
		Ethidium bromide displacement fluorescence assay	25 μg/mL (107.30 μM)	DNA intercalation (27%)	AsPC-1	IC_50_ (72 h): >21.46 μM	↓ Cell cycle in phase G2	
					BxPC-3	IC_50_ (72 h): >21.46 μM		
					Mia PaCa2			
					Vero^d^			
Makaluvamine A	*Zyzzya* cf. *marsailis*	Cell-free decatenation reaction of kinetoplast DNA	IC_90_: 41 μM	↓ DNA relaxation	HCT-116	IC_50_ (time not indicated): 1.3 μM		[49]
		Cell-free DNA cleavage assay with supercoiled pBR322 DNA plasmid	IC_50_: 2.1 μM	Topo II-mediated cleavage of plasmid DNA	CHO xrs-6 ^c^	IC_50_ (time not indicated): 0.41 μM		
		Neutral filter elution assay	Not indicated	Production of cleavable complexes (strand scission factor = 1.38)	Balb/C nu/nu athymic mice bearing OVCAR-3 xenograft		↓ Tumor mass: T/C: 62% (0.5 mg/kg for 4 weeks)	
		Analysis of absorbance spectra of calf thymus DNA	Not indicated	53% absorption hypochromism				
	*Zyzzya* *fuliginosa*	Cell-free DNA cleavage assay with radiolabeled and supercoiled rf M13 mp19 DNA plasmid	91 mM	17% of topo II-mediated cleavage of plasmid DNA (compared to 100% of etoposide)				[51]
Makaluvamine B	*Zyzzya* cf. *marsailis*	Cell-free decatenation reaction of kinetoplast DNA	IC_90_: 500 μM	↓ DNA relaxation	HCT-116	IC_50_ (time not indicated): >50 μM		[49]
		Cell-free DNA cleavage assay with supercoiled pBR322 DNA plasmid	IC_50_: 181 μM	Topo II-mediated cleavage of plasmid DNA	CHO xrs-6 ^c^	IC_50_ (time not indicated): 13.49 μM		
Makaluvamine C	*Zyzzya* cf. *marsailis*	Cell-free decatenation reaction of kinetoplast DNA	IC_90_: 420 μM	↓ DNA relaxation	HCT-116	IC_50_ (time not indicated): 36.2 μM	/	[49]
		in vitro cell-free DNA cleavage assay with supercoiled pBR322 DNA plasmid	IC_50_: 1.2 μM	Topo II-mediated cleavage of plasmid DNA				
		Analysis of absorbance spectra of calf thymus DNA	Not indicated	66% absorption hypochromism	CHO xrs-6 ^c^	IC_50_ (time not indicated): 5.4 μM		
					Balb/C nu/nu athymic mice bearing OVCAR-3 xenograft		↓ Tumor mass: T/C: 48% (5 mg/kg for 4 weeks)	
					Immune competent mice inoculated with P388 ^e^ cells		↑ MLS: 18% (5 mg/kg for 4 weeks)	
	*Zyzzya * *fuliginosa*	Cell-free DNA cleavage assay with radiolabeled and supercoiled rf M13 mp19 DNA plasmid and human topo II	91 mM	16% of topo II-mediated cleavage of plasmid DNA (compared to 100% of etoposide)				[51]
		Cell-free cleavage assay of pUC 19 radiolabeled DNA with human topo II	33–466 μM	Cleavage of pUC 19 DNA at nucleoside A329 Formation of cleavable complex				
Makaluvamine D	*Zyzzya* cf. *marsailis*	Cell-free decatenation reaction of kinetoplast DNA	IC_90_: 320 μM	↓ DNA relaxation	HCT-116	IC_50_ (time not indicated): 17.1 μM		[49]
		Cell-free DNA cleavage assay with supercoiled pBR322 DNA plasmid	IC_50_: 52 μM	Topo II-mediated cleavage of plasmid DNA	CHO xrs-6 ^c^	IC_50_ (time not indicated): 14 μM		
	*Zyzzya* *fuliginosa*	Cell-free DNA cleavage assay with radiolabeled and supercoiled rf M13 mp19 DNA plasmid and topo II	91 mM	5% of topo II-mediated cleavage of plasmid DNA (compared to 100% of etoposide)				[51]
		in vitro cell-free cleavage assay of pUC 19 radiolabeled DNA with human topo II	33–466 μM	Cleavage of pUC 19 DNA at nucleoside A329 Formation of cleavable complex				
Makaluvamine E	*Zyzzya* cf. *marsailis*	Cell-free decatenation reaction of kinetoplast DNA	IC_90_: 310 μM	↓ DNA relaxation	HCT-116	IC_50_ (time not indicated): 1.2 μM		[49]
		Cell-free DNA cleavage assay with supercoiled pBR322 DNA plasmid	IC_50_: 15 μM	Topo II-mediated cleavage of plasmid DNA	CHO xrs-6 ^c^	IC_50_ (time not indicated): 1.7 μM		
	*Zyzzya fuliginosa*	Cell-free DNA cleavage assay with radiolabeled and supercoiled rf M13 mp19 DNA plasmid and topo II	91 mM	22% of topo II-mediated cleavage of plasmid DNA (compared to 100% of etoposide)				[51]
		Cell-free cleavage assay of pUC 19 radiolabeled DNA with human topo II	33–466 μM	Cleavage of pUC 19 DNA at nucleoside A329 Formation of cleavable complex				
Makaluvamine F	*Zyzzya* cf. *marsailis*	Cell-free decatenation reaction of kinetoplast DNA	IC_90_: 25 μM	↓ DNA relaxation	HCT-116	IC_50_ (time not indicated): 0.17 μM		[49]
		Cell-free DNA cleavage assay with supercoiled pBR322 DNA plasmid	IC_50_: 1.1 μM	Topo II-mediated cleavage of plasmid DNA	CHO xrs-6 ^c^	IC_50_ (time not indicated): 0.08 μM		
Makaluvamine H	*Zyzzya* *fuliginosa*	Cell-free DNA cleavage assay with radiolabeled and supercoiled rf M13 mp19 DNA plasmid and topo II	91 mM	33% of topo II-mediated cleavage of plasmid DNA (compared to 100% of etoposide)	Athymic nude mice bearing KB xenograft	Not indicated	↓ Tumor growth (22 mg/kg, days 1, 4, and 8 for 28 days)	[51]
Makaluvamine I	*Zyzzya* *fuliginosa*	Cell-free DNA cleavage assay with radiolabeled and supercoiled rf M13 mp19 DNA plasmid and topo II	91 mM	61% of Topo II-mediated cleavage of plasmid DNA (compared to 100% of etoposide)	CHO xrs-6 ^c^	IC_50_ (time not indicated): 0.4 μM		[51]
					CHO AA8 ^c^	IC_50_ (time not indicated): 2 μM		
					Athymic nude mice bearing KB xenograft		↓ Tumor growth (11 mg/kg, days 1, 4, and 8 for 28 days)	
Makaluvamine N	*Zyzzya* *fuliginosa*	Cell-free DNA relaxation assay using a supercoiled pBR322 DNA plasmid and human topo II	/	↓ Topo II unwinding (>90% at 5 μg/mL)	HCT-116	IC_50_ (72 h): 0.6 μg/mL		[46]
	*Zyzzya* *fuliginosa*	Cell-free DNA cleavage assay with radiolabeled supercoiled rf M13 mp19 DNA plasmid and topo II	91 mM	26% of topo II-mediated cleavage of plasmid DNA (compared to 100% of etoposide)				[51]
Makaluvamine V	*Zyzzya fuliginosa*	Cell-free DNA cleavage assay with radiolabeled supercoiled rf M13 mp19 DNA plasmid and topo II	91 mM	2% of topo II-mediated cleavage of plasmid DNA (compared to 100% of etoposide)				[51]
Manoalide 25-acetals	*Hirtios erecta*	Not indicated	IC_50_: 25 μM	↓ DNA-unknotting activity of calf thymus DNA topo II	CDF_1_ mice inoculated with P388 ^e^ cells		T/C: 150% (1 μg/g)	[60]
Neoamphidemine	*Xestospongia* sp.	Cell-free decatenation reaction of kinetoplast DNA	0.5–30 μM	↓ DNA decatenation	HEK-293	IC_50_ (72 h): 0.8 μM		[29]
		Malachite green assay with supercoiled DNA, recombinant human topoIIα	0–10 μM	Competitive inhibition of topo II-mediated ATP hydrolysis	HEK293-Metnase	IC_50_ (72 h): 0.5 μM		
		Molecular docking	/	Bind to the ATPase site of topo II				
		Not indicated	Not indicated	Catenation of DNA to high molecular weight complex	CHO AA8 ^c^	IC_50_ (time not indicated): 2 μg/mL		[27]
		Not indicated	Not indicated	3% of topo II-mediated DNA cleavage				
		Cell-free DNA cleavage assay using radiolabeled and supercoiled rf M13 mp19 DNA and human topo II	Not indicated	Catenation of DNA to high molecular weight complex	HCT-116	IC_50_ (72 h): 4.5 μM		[25]
			50 μM	8.9% of topo II-mediated DNA cleavage				
		Transmission electron microscopy analysis	Not indicated	Catenation of DNA	SK-mel-5	IC_50_ (72 h): 7.6 μM		
		DNA filter-binding assay	100–600 μM	Catenation of DNA through DNA aggregation	KB	IC_50_ (72 h): 6 μM		
					MCF-7	IC_50_ (72 h): 1.8 μM		
					A2780	IC_50_ (72 h): 0.9 μM		
					A2780AD	IC_50_ (72 h): 0.83 μM		
					CHO AA8 ^c^	IC_50_ (72 h): 2.5 μM		
					CHO xrs-6 ^c^	IC_50_ (72 h): 1.6 μM		
					Balb/c nu/nu mice bearing HCT-116 xenograft		↓ Tumor growth (12.5, 25, and 50 mg/kg for 19 days)	
					Balb/c nu/nu mice bearing KB xenograft		↓ Tumor growth (50 mg/kg for 19 days)	
Popolohuanone E	*Dysidea* sp.	Not indicated	IC_50_: 400 μM	↓ Topo II activity	P388 ^e^	IC_50_: 20 μg/mL		[81]
					HT-29	IC_50_: >20 μg/mL		
					A549	IC_50_: 2.5 μg/mL		
					CV-1 ^d^	IC_50_: >20 μg/mL		
Secobatzelline A	*Batzella* sp.	Cell-free decatenation reaction of kinetoplast DNA	25 μg/mL (98.04 μM)	↓ DNA decatenation (61%)	Panc-1	IC_50_ (72 h): 10.39 μM		[54]
		Ethidium bromide displacement fluorescence assay	25 μg/mL (98.04 μM)	DNA intercalation (34%)	AsPC-1	IC_50_ (72 h): 3.62 μM	↓ Cell cycle in phase S	
					BxPC-3	IC50 (72 h): 4.10 μM		
					Mia PaCa2	IC_50_ (72 h): 5.62 μM		
					Vero^d^	IC_50_ (72 h): 14.03 μM		
Secobatzelline B	*Batzella* sp.	Cell-free decatenation reaction of kinetoplast DNA	25 μg/mL (97.66 μM)	↓ DNA decatenation (13%)	Panc-1	IC_50_ (72 h): 17.38 μM		[54]
		Ethidium bromide displacement fluorescence assay	25 μg/mL (97.66 μM)	DNA intercalation (17%)	AsPC-1	IC_50_ (72 h): >19.531 μM	↓ Cell cycle in phase S	
					BxPC-3	IC_50_ (72 h): >19.53 μM		
					Mia PaCa2			
					Vero^d^			
Secoadociaquinone A	*Xestopongia* sp.	Cell-free decatenation reaction of kinetoplast DNA	IC_90_: 113 μM	↓ DNA decatenation	HCT-116	IC_50_ (18 + 72 h): >143 μM		[77]
					CHO xrs-6 ^c^	IC_50_ (18 + 72 h): >247 μM		
Secoadociaquinone B	*Xestopongia* sp.	Cell-free decatenation reaction of kinetoplast DNA	IC_90_: 113 μM	↓ DNA decatenation	HCT-116	IC_50_ (18 + 72 h): >143 μM		[77]
					CHO xrs-6 ^c^	IC_50_ (18 + 72 h): >247 μM		
Xestoquinone	*Petrosia* sp.	Cell-free DNA cleavage assay using an enzyme-mediated negatively-supercoiled pHOT1 plasmid DNA and topo II	IC_50_: 0.094 μM	↓ DNA relaxation	Molt-4	IC_50_ (24 h): 2.95 μM	Apoptosis (↓ MMP; ↑ cleaved PARP, ↑ cleaved caspase-3, ↑ cleaved caspase-7, ↑ cleaved caspase-8, ↑ cleaved caspase-9, ↑ Bax, ↑ Bak, ↑ cyt c, ↑ Fas, ↑ TRADD, ↓ Bcl-2, ↓ Bid, ↓ XIAP protein expression ↑ ER stress (↑ Ca^2+^ release; ↑ CHOP, ↓ IRE 1α, ↓ PERK protein expression) ↓ HDAC activity (↓ HDAC1, ↓ HDAC3, ↓ HDAC4, ↓ HDAC6, ↓ HDAC7, ↓ HDAC8 protein expression) DNA damage (↑ p-Chk1, ↑ p-Chk2, ↑ γH2AX protein expression) Oxidative stress (↑ ROS) Interaction with Hsp90	[76]
					Immunodeficient athymic mice bearing Molt-4 xenograft		↓ Tumor growth, ↓ tumor weight, ↓ tumor volume (1μg/g/day for 50 days) ↓ HDAC1, ↓ HDAC3, ↓ HDAC8 protein expression	
		Western Blotting on Molt-4 and K562 cells	7.84 μM	↓ Topo IIα protein expression	K562	IC_50_ (24 h): 6.22 μM		
					Sup-T1	IC_50_ (24 h): 8.58 μM		
					U937	IC_50_ (24 h): 11.12 μM		
					NR8383 ^d^	IC_50_ (24 h): >30 μM		

↑: upregulation/induction; ↓: downregulation/inhibition; ^a^: concentration that inhibits 50% or 90% of topo II activity; ^b^: concentration that inhibits 50% of cell viability; ^c^: Chinese hamster ovary (CHO) double strand break repair-deficient cells; ^d^: non tumor cells; ^e^: murine cancer cells; A2780AD: A2780 multidrug resistant cells; Akt: protein kinase B; ATF6: activating transcription factor 6; ATM: ataxia telangiectasia mutated; ATR: ATM and RAD3-related; Bax: BCL2-associated X protein; Bcl-2: B-cell lymphoma 2; Ca^2+:^ calcium; CDK4: cyclin-dependent kinase 4; Chk1: checkpoint kinase 1; Chk2: checkpoint kinase 2; Cyt c: cytochrome c; CHOP: C/EBP homologous protein; DSBs: double-strand breaks; EGFR: epidermal growth factor receptor; EMT: epithelial-mesenchymal transition; ER: endoplasmic reticulum; ERK: extracellular signal-regulated kinase; GSK 3β: glycogen synthase kinase-3 β; GRP4: glucose-regulated protein 94; H3: histone H3; HDAC: histone deacetylase; HK: hexokinase; HIF 1: *hypoxia-inducible factor 1*; *HIF*-*1α*: *hypoxia-inducible factor 1*-*alpha*; HO-1: heme oxygenase 1; HSF1: heat shock transcription factor 1; Hsp70: heat shock protein 70; Hsp90: heat shock protein 90; IRAK1: interleukin-1 receptor-associated kinase 1; IRE 1α: inositol-requiring enzyme 1α; JNK: Jun N-terminal kinase; KSDS: potassium sodium dodecyl sulfate; LC3-II: microtubule-associated proteins 1A/1B light chain 3B, LC3-phosphatidylethanolamine conjugate; mTOR: mammalian target of rapamycin; MDM 2: murine double minute 2; MLS: median life span; MMP: mitochondrial membrane permeabilization; MnSOD: manganese superoxide dismutase; NFκB: nuclear factor kappa B; NOX: NADPH oxidase; p-: phosphorylated; PARP: poly (ADP-ribose) polymerase; PCNA: proliferating cell nuclear antigen; PDK1: 3-phosphoinositide-dependent kinase 1; PERK: protein kinase RNA-like endoplasmic reticulum kinase; PKM2: pyruvate kinase muscle isozyme M2; PTEN: phosphatase and tensin homolog; Raf-1: V-raf-1 murine leukemia viral oncogene homolog 1; ROS: reactive oxygen species; Src: proto-oncogene tyrosine-protein kinase; STAT3: signal transducers and activators of transcription 3; T/C: ratio between the tumor volume in the treated (T) group and in the untreated control (C) group; TRADD: Fas-associated death domain protein; XIAP: X-linked inhibitor of apoptosis protein. Human breast cancer cell lines: MCF-7; MDA-MB-231; T-47D. Human cervical cancer cell line: HeLa. Human colon cancer cell lines: DLD-1; HCT-116; HT-29. Human epithelial carcinoma cell line: KB. Human gastric cancer cell line: NUGC-3. Human leukemia cell lines: MOLT-4; K562; HL-60. Human lymphoma cell lines: U937; Sup-T1. Human lung cancer cell line: A549. Human melanoma cell lines: MEL-28; SK-mel-5. Human ovarian cancer cell lines: OVCAR-3; A2780. Human oral carcinoma cell lines: Ca9-22; Cal-27. Human pancreatic cancer cell lines: Panc-1; AsPC-1; BxPC-3; Mia PaCa2. Human prostate cancer cell lines: LnCap; PC-3; Du-145.

## 3. Topo II Inhibitors from Marine Fungi and Bacteria

### 3.1. Leptosin F

**Figure 14:**
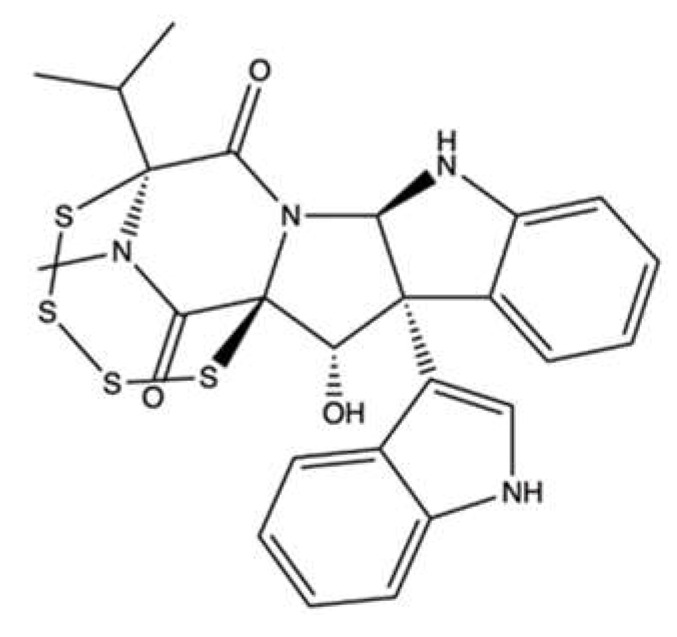
Chemical structure of leptosin F (CAS number: not available).

Leptosin F (**LEP**, Figure 14) is an indole derivative containing sulphur that is derived from the fungus *Leptoshaeria* sp., which grows on the marine alga *Sargassum tortile* [82].

Yanagihara and colleagues demonstrated that **LEP** potently inhibited the growth of RPMI-8402 T cell acute lymphoblastic leukemia cells–more powerfully than ETO and with an IC_50_ value in the nM range–and induced apoptosis [82]. A pro-apoptotic effect has also been reported for **LEP** in normal human embryo kidney cells (293 cell line), where it activated Casp-3 at doses as low as 1 to 10 µM [82]. These results could indicate that **LEP** does not act selectively against cancer cells, but rather on all rapidly proliferating cells. 

The in vitro kDNA decatenation assay revealed its ability to inhibit topo II [82]. Gel electrophoresis of the kDNA after decatenation assay showed that **LEP** did not act as a catalytic inhibitor of topo II, as the authors instead stated. Further studies would be necessary to define the exact mechanism of interaction between **LEP** and the enzyme. Moreover, since the compound concentration required to exert cytotoxic activity on RPMI-8402 cells was extremely lower (nM range) than that required to inhibit topo II (µM range), the cytotoxicity of **LEP** at the cellular level might involve other pathways in addition to the inhibition of topo II.

### 3.2. Pericosine A

**Figure 15:**
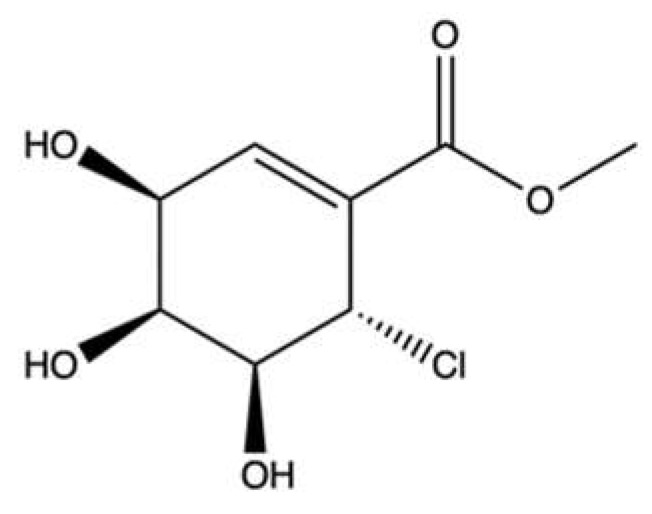
Chemical structure of pericosine A (CAS number: 200335-68-8).

Pericosine A (**PA**, Figure 15) is a metabolite produced by a strain of *Periconia byssoides* OUPS-N133, a marine fungus originally separated from the sea hare *Aplysia kurodai* [83]. 

Some studies reported the ability of **PA** to induce growth inhibition on different cancer cell lines [83,84] (Table 2). Furthermore, in mice inoculated with P388 leukemic cells, **PA** increased the median survival days compared to vehicle (13.0 versus 10.7 days) (Table 2). In the same study, the authors reported that **PA** at 100–300 mM inhibited topo II and at 449 μM inhibited the epidermal growth factor receptor (EGFR) by 40−70%. Since **PA** seems to exert its inhibitory effects on topo II at very high concentrations, it is unlikely that this mechanism of action was responsible for its in vitro and in vivo antitumor effects. The inhibition of EGFR, a protein kinase known to promote cell proliferation and counteract apoptosis [85], could be a more plausible mechanism [83]. The lack of important information on its antitumor activity in vitro and in vivo does not permit a clear characterization of the anticancer activity of **PA**. Therefore, further experiments should be conducted to fully understand the potential usefulness of **PA** in the oncological area. 

### 3.3. Marinactinone B

**Figure 16:**
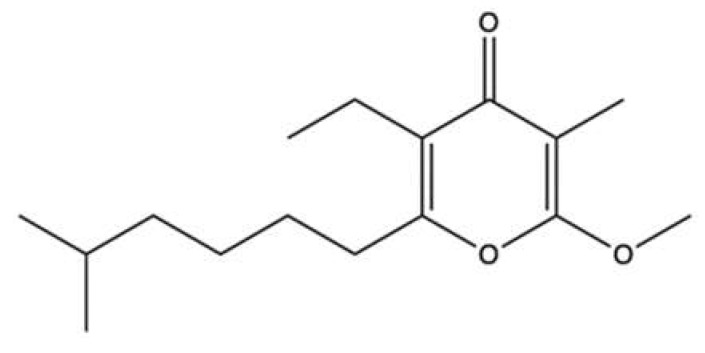
Chemical structure of marinactinone B (CAS number: 1344677-16-2).

Marinactinone B (**MB**, Figure 16) is a γ-pyrone derivate isolated from the bacterial strain *Marinactinospora thermotolerans* SCSIO 00606, found in the sediments of the northern South China Sea [86].

**MB** was evaluated for its anticancer activity against breast (MCF-7), pancreatic (SW1990), hepatic (HepG2 and SMCC-7721), lung (NCI-H460), and cervical (HeLa) cancer cell lines. It exhibited cytotoxicity at medium-elevated concentration values only against SW1990 (99 µM) and SMCC-7721 (45 µM) cell lines. It was also a very weak inhibitor of topo II with an IC_50_ value of 607 µM [86]. With such a high IC_50_ value, **MB** is not a promising compound per se. However, given its interaction with topo II, **MB** could constitute the basis for the development of analogues with antitumor activity.

### 3.4. Aspergiolide A

**Figure 17:**
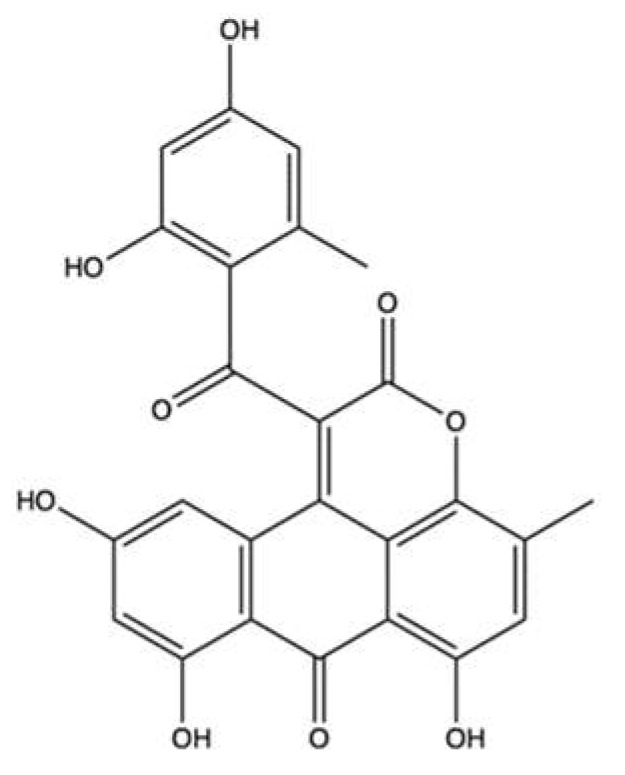
Chemical structure of aspergiolide A (CAS number: 915160-58-6).

Aspergiolide A (**ASP**, Figure 17) is an anthracycline [87] isolated from *Aspergillus glaucus*, which was obtained from the marine sediment around mangrove roots harvested in the Chinese province of Fujian [88].

**ASP** was cytotoxic on different human and murine cancer cell lines (Table 2) [88]. 

Wang et al. have delved into the antitumor efficacy of **ASP** in vitro and in vivo. The compound induced Casp-dependent apoptosis as early as 12 h after treatment [87]. In addition, **ASP** increased γ-H2A.X protein expression. Considering its anthracyclinic structure, it has been hypothesized that the inhibition of topo II could be involved in its apoptotic activty. The kDNA decatenation assay demonstrated that **ASP** inhibited the enzyme in a fashion comparable to DOXO. The results of in vivo experiments in H22 hepatoma-bearing mice and on BEL-7402 cancer xenografts (Table 2) corroborated the in vitro findings. **ASP** reduced tumor volume dose-dependently in H22 mice and showed comparable activity to that of DOXO (2 mg/kg). In BEL-7402 xenografts, **ASP** showed significantly milder activity than DOXO. Interestingly, in both in vivo models, **ASP** altered mice body weight considerably less than DOXO, suggesting less toxicity than the benchmark anthracycline [87]. The study also investigated the pharmacokinetic profile of **ASP**, which has been shown to distribute throughout the body in a perfusion- and blood-flow-dependent manner, and was able to concentrate in tumor tissues. Additionally, **ASP** penetrated the blood brain barrier. No clinical signs of toxicity or organs morphological changes were found in mice treated with the maximal tolerable dose of **ASP** (more than 400 mg/kg) [87], which is considerably higher than the dose necessary to produce the antitumor effects. The genotoxic potential of **ASP** was also evaluated via the in vivo bone marrow erythrocyte micronucleus assay. The number of micronuclei produced following treatment with **ASP** was comparable to the negative control, suggesting that **ASP** was not genotoxic [87]. 

Anthracyclines are proven to cause significant cardiotoxicity and electrocardiogram abnormalities including long QT syndrome, a potentially lethal condition induced by several drugs [89]. Long QT syndrome has been found to be caused by the blockade of hERG (human ether-a-go-go-related gene), a gene codifying the pore-forming subunit of the potassium channels, which are relevant for cardiac repolarization [90]. Thus, Li et al. investigated the in vitro inhibitory rates of **ASP** on the hERG current. The resulting values indicated that **ASP** was unable to inhibit the hERG channel, and hence it is unlikely to produce cardiotoxicity through this mechanism [87]. 

On the whole, the studies reported above identify **ASP** as an attractive candidate in the oncological area. However, further studies will be necessary to clarify whether the effects of the compound can be attributed to topo II inhibition. 

### 3.5. Jadomycin DS

**Figure 18:**
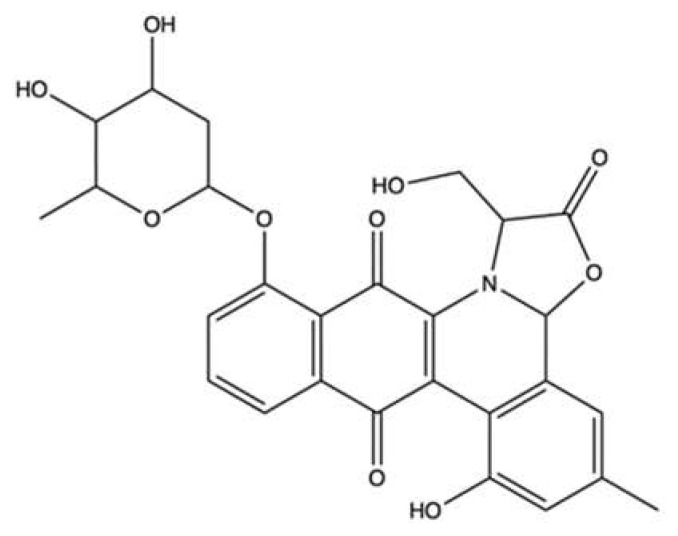
Chemical structure of jadomycin DS (CAS number: not available).

Jadomycin DS (**JAD**, Figure 18) is a polyketide produced by the bacterium *Streptomyces venezuelae* ISP5230 under stress conditions [91].

**JAD** shares three common features with ETO and DOXO: (i) a lactone ring, (ii) a quinone moiety, and (iii) a copper-mediated DNA cleavage activity. To estimate the molecular interactions of **JAD**, binding studies were conducted using a nuclear magnetic resonance spectroscopy (NMR) method that allows the identification of molecules capable of binding a ligand-protein with binding affinity (K_D_) in the μM−mM range [92,93]. **JAD** bound topo IIβ. However, the overall K_D_ for **JAD**-topo IIβ complex was equal to 9.4 mM, suggesting that the bond formed between **JAD** and topo IIβ is weak [91]. The high binding constant between the compound and topo IIβ does not depict **JAD** as an attractive anti-cancer drug. Moreover, **JAD** interacted unselectively with several unrelated enzymes including serum albumin [91], making it difficult to determine its actual mode of action and severely compromise its hypothetic in vivo application. 

### 3.6. 2R-Acetoxymethyl-1,3,3-trimethyl-4t-(3-methyl-2-buten-1-yl)-1t-cyclohexanol

**Figure 19:**
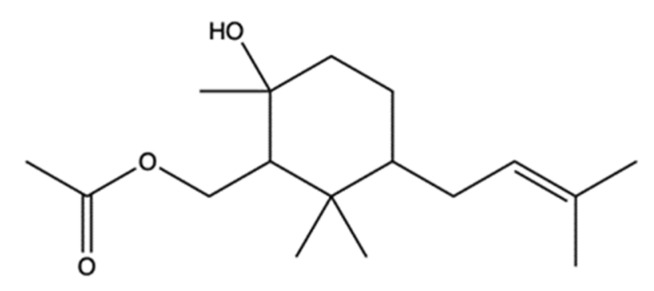
Chemical structure of 2R-acetoxymethyl-1,3,3-trimethyl-4t-(3-methyl-2-buten-1-yl)-1t-cyclohexanol (CAS number: not available).

2R-acetoxymethyl-1,3,3-trimethyl-4t-(3-methyl-2-buten-1-yl)-1t-cyclohexanol (**2RA**, Figure 19) is a sesquiterpene derived from *Streptomyces* sp. VITJS8 found in Indian marine soil [94].

**2RA** was cytotoxic [94], blocked the cell cycle in the G2/M phase, and triggered Casp-dependent apoptosis in HepG2 cells. To determine whether **2RA** was able to interact with human topo IIα, a molecular docking study was performed, demonstrating that **2RA** was able to bind to the active receptor pocket with a binding energy of −7.84 kJ/mol [94]. In addition, an increased formation of hydrogen bonds in the protein–ligand complex was recorded compared to the protein, indicating that the protein–ligand complex had a higher binding affinity and stability than the protein [94]. However, in vitro studies should be conducted to demonstrate that **2RA** is a topo II α inhibitor.

### 3.7. Streptomyces *sp.* VITJS4 Ethyl Acetate Crude Extract

*Streptomyces* sp. VITJS4 bacterial strain was isolated from the marine environment in Tamil Nadu, India [95]. VITJS4 ethyl acetate crude extract exerted cytotoxic effects against HepG2 and HeLa cancer cells with identical IC_50_ values of 50 μg/mL and induction of apoptosis. Hence, this would suggest a cell line-independent mechanism of action [95]. Gas chromatography–mass spectrum analysis (GC–MS) identified a phthalate derivative, namely 1, 2-benzenedicarboxylic acid, mono- (2-ethylhexyl) ester, as the major bioactive metabolite among the 52 bioactive compounds of the ethyl acetate extract, which is probably responsible for the activity observed on the two human cancer cell lines. Molecular docking analysis was conducted to assess the interaction between the compound and topo IIα. What emerged is the formation of bonds at the active pocket of protein with a binding energy of −5.87 kJ/mol [95].

### 3.8. Sulochrin

**Figure 20:**
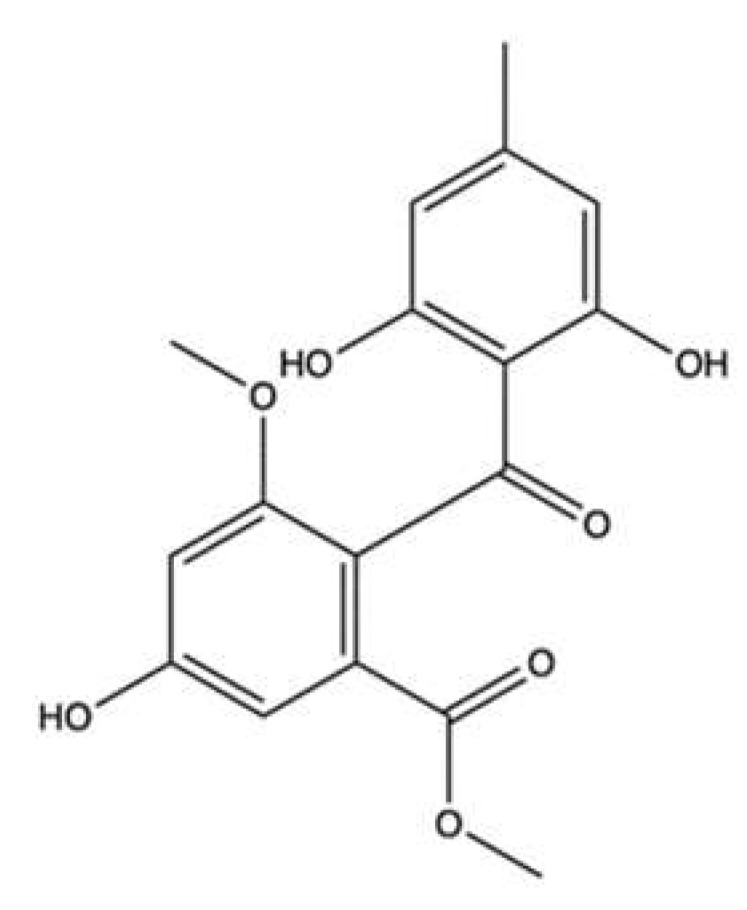
Chemical structure of sulochrin (CAS number: 519-57-3).

Sulochrin (Figure 20) is a benzophenone derivative isolated from *Aspergillus falconensis* after cultivating it on a solid rice medium containing 3.5% of (NH_4_)_2_SO_4_ [96]. 

Sulochrin was cytotoxic on L5178Y murine lymphoma cell line with an IC_50_ value of 5.1 µM [96]. The compound was not cytotoxic on MDA-MB-231 human breast cancer cells; however, at a concentration of 70 µM, it dramatically reduced cell migration [96]. Molecular docking studies indicated the interaction of sulochrin with topo II. With a free binding energy of −12.11 kcal/mol, the compound showed a robust stability through the formation of several stable bonds within the active sites, comparable to that exerted by DOXO (−16.28 kcal/mol). Molecular docking studies also demonstrated the capacity of the compound to even bind within the active sites of two further enzymes: the cyclin-dependent kinase 2 (CDK2) involved in cell-cycle progression, and the matrix metalloproteinase 13 (MMP-13) involved in the EMT process, with moderate free binding energies [96]. 

### 3.9. 3-Hydroxyholyrine A

**Figure 21:**
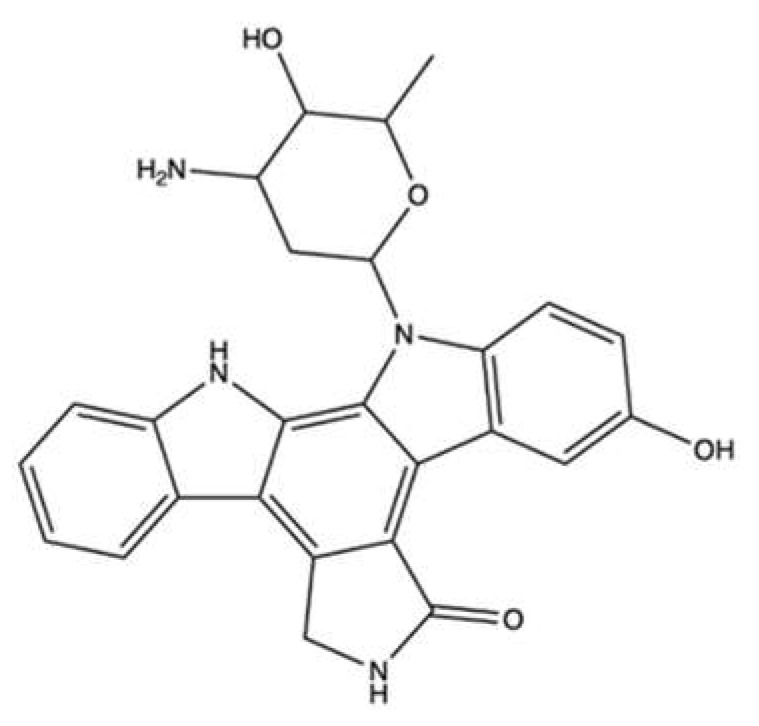
Chemical structure of 3-hydroxyholyrine A (CAS number: 2226941-28-0).

3-hydroxyholyrine A (**3HA**, Figure 21) is an indolocarbazole produced by the marine-derived bacterium *Streptomyces* strain OUCMDZ-3118 in the presence of 5-hydroxy-L-tryptophan [97].

**3HA** exerted cytotoxic effects on many tumor cell lines (Table 2) and reduced the expression of the antiapoptotic protein survivin more potently than ETO in MKN45 cells [97]. In supercoiled plasmid DNA relaxation assay, **3HA** potently inhibited the activity of topo IIα enzyme at 1.0, 5.0, and 10.0 μM. Of note, **3HA** exhibited an inhibitory activity at concentrations lower than ETO (50 μM). The inhibition of topo IIα resulted in DNA damage, as demonstrated by the concentration-dependent increase in the expression of γ-H2A.X.

**Table 2:** Topo II inhibitors derived from marine fungi and bacteria.

Compound	Source	Topo II Inhibitory Activity			Antitumor Effect(s)			Ref.
		Assay	Concentration(s) Tested or IC_50_	Outcomes	Experimental Model	Cytotoxic Activity	Other Antitumor Mechanism(s)	
2R-acetoxymethyl-1,3,3-trimethyl-4t-(3-methyl-2-buten-1-yl)-1t-cyclohexanol	Bacterium *Streptomyces* sp. VITJS8	Molecular docking			HepG2	IC_50_ (16 h): 250 µg/mL	Apoptosis (caspase-9, caspase-8, caspase-3 cleavage, regulation of Bcl-2 family proteins, cell shrinkage, chromatin condensation, apoptotic bodies, DNA fragmentation, incomplete nuclear membrane) ↓ Cell growth (cell cycle arrest: ↑ cells in S and G2/M phases, ↓ cells in G0/G1 phase)	[94]
3-hydroxyholyrine A	Bacterium *Streptomyces* strain OUCMDZ-3118	Cell free DNA relaxation assay using supercoiled pBR322 DNA plasmid and topo II α	Not indicated		A-549	IC_50_ (48 h): 0.51 µM		[97]
					MCF-7	IC_50_ (48 h): 5.0 µM		
					K562	IC_50_ (48 h): 7.2 µM		
					AGS	IC_50_ (48 h): 1.7 µM	Apoptosis (↓ survivin protein expression) DNA damage (↑ γ-H2AX protein expression)	
					MKN45	IC_50_ (48 h): 4.3 µM	Apoptosis (↓ survivin protein expression)	
Aspergiolide A	Fungus *Aspergillus glaucus*				A-549	IC_50_ (24 h): 0.13 µM		[88]
					HL-60	IC_50_ (72 h): 0.28 µM		
					BEL-7402	IC_50_ (24 h): 7.5 µM		
					P388 ^f^	IC_50_ (72 h): 35.0 µM		
		Spectrofluorimetric decatenation reaction of kinetoplast DNA	10–100 µM	↓ Topo II activity	HeLa	IC_50_ (72 h): 2.37–7.07 µM		[87]
					SMMC-7721			
					SGC-7901			
					MCF-7			
					MDA-MB-468			
					U251			
					A431			
					SK-OV-3			
					BxPC-3			
					786-O			
					BEL-7402	IC_50_ (72 h): 2.37–7.07 µM	Apoptosis (procaspase-3, procaspase-8, procaspase-9 and PARP cleavage, ↑ Bax protein expression, ↓ Bcl-2 protein expression) DNA damage (↑ γ-H2AX protein expression)	
					KN mice inoculated with H22 ^f^ cells		↓ Tumor growth (5, 15, 45 mg/kg i.p.)	
					Nude mice bearing BEL-7402 xenografts		↓ Tumor volume (7, 14, 28 mg/kg/day i.p. for 21 days)	
Jadomycin DS	Bacterium *Streptomyces venezuelae* ISP5230	WaterLOGSY NMR spectroscopy		Interaction with topo IIβ				[91]
Leptosin F	Fungus *Lestoshaeria* sp.	Cell-free decatenation reaction of kinetoplast st DNA	10–30 µM	↓ Topo II activity	RPMI8402	IC_50_ (72 h): 8.2 nM	Apoptosis (↑ caspase-3 activity, ↑ DNA degradation)	[82]
					293 ^d^		Apoptosis (caspase-3 activation Akt inactivation)	
					P388 ^f^	ED_50_: 0.056 µg/cm^3^		[98]
Marinactinone B	Bacterium *Marinactinospora thermotolerans*	Cell free relaxation assay using supercoiled pBV220 DNA plasmid and topo II from rat liver cells	607 µM	↓ Topo II activity	SW1990	IC_50_ (72 h): 99 µM		[86]
Pericosine A	Fungus *Periconia byssoides*		100–300 mM	↓ Topo II activity	P388 ^f^	ED_50_: 0.1 µg/mL		[83]
					HBC-4	Log GI_50_/M: -4.76		
					BSY-1	Log GI_50_/M: -4.75		
					HBC-5	Log GI_50_/M: -5.22		
					MCF-7	Log GI_50_/M: -4.66		
					MDA-MB-231	Log GI_50_/M: -4.74		
					U-251	Log GI_50_/M: -4.76		
					SF-268	Log GI_50_/M: -4.72		
					SF-295	Log GI_50_/M: -4.62		
					SF-539	Log GI_50_/M: -4.71		
					SNB-75	Log GI_50_/M: -7.27		
					SNB-78	Log GI_50_/M: -4.71		
					HCC2998	Log GI_50_/M: -4.75		
					KM-12	Log GI_50_/M: -4.73		
					HT-29	Log GI_50_/M: -4.70		
					WiDr	Log GI_50_/M: -4.64		
					HCT-15	Log GI_50_/M: -4.77		
					HCT-116	Log GI_50_/M: -4.75		
					NCI-H23	Log GI_50_/M: -4.78		
					NCI-H226	Log GI_50_/M: -4.80		
					NCI-H522	Log GI_50_/M: -4.95		
					NCI-H460	Log GI_50_/M: -4.72		
					A-549	Log GI_50_/M: -4.61		
					DMS273	Log GI_50_/M: -4.68		
					DMS114	Log GI_50_/M: -4.82		
					LOX-IMVI	Log GI_50_/M: -4.72		
					OVCAR-3	Log GI_50_/M: -4.85		
					OVCAR-4	Log GI_50_/M: -4.68		
					OVCAR-5	Log GI_50_/M: -4.79		
					OVCAR-8	Log GI_50_/M: -4.78		
					SK-OV-3	Log GI_50_/M: -4.76		
					RXF-631L	Log GI_50_/M: -4.73		
					ACHN	Log GI_50_/M: -4.72		
					St-4	Log GI_50_/M: -4.65		
					MKN1	Log GI_50_/M: -4.78		
					MKN7	Log GI_50_/M: -4.70		
					MKN28	Log GI_50_/M: -4.72		
					MKN45	Log GI_50_/M: -4.75		
					MKN74	Log GI_50_/M: -4.69		
					Mice inoculated with P388 ^f^ cells		↑ Survival days compared to controls (25 mg/kg administered i.p. on day 1 and 5)	
*Streptomyces* sp. VITJS4 strain crude extract	Bacterium *Streptomyces* sp. VITJS4	Molecular docking			HeLa	IC_50_ (time not indicated): 50 µg/mL	Apoptosis DNA damage (stained nuclei with round morphology, chromatin condensation, DNA fragmentation)	[95]
					HepG2	IC_50_ (time not indicated): 50 µg/mL		
Sulochrin	Fungus *Aspergillus falconensis*	Molecular docking			L5178Y ^f^	IC_50_ (24 h): 5.1 µM		[96]
					MDA-MB-231	No cytotoxic activity	↓ Cell migration	

↑: upregulation/induction; ↓: downregulation/inhibition; ^d^ non-transformed cells; ^f^ murine cancer cells; Akt: protein kinase B; Bax: Bcl-2 associated X protein; Bcl-2: B-cell lymphoma 2; ED_50_: dose effective in 50% of treated subjects; γ-H2AX: phosphorylated H2A histone family member X; IC_50_: concentration that inhibits 50% of the investigated activity; i.p.: intraperitoneal; Log GI_50_: logarithm of concentration that inhibits 50% of cell growth; PARP: poly (ADP-ribose) polymerase. Human breast cancer cell lines: MCF-7; MDA-MB-231; MDA-MB-468; BSY-1. Human cervical cancer cell line: HeLa. Human colon cancer cell lines: HCT-116; HT-29; HCT2998; KM-12; WiDr; HCT-15. Human epidermoid cancer cell line: A431. Human gastric cancer cell line: AGS; MKN45; SGC-7901; St-4; MKN1; MKN7; MKN28; MKN74. Human glioma cell lines: U251. Human glioblastoma cell lines: SF-268; SF-295; SF-539; SNB-75; SNB-78. Human hepatic cancer cell lines: HepG2; BEL-7402; SMMC-7721. Human leukemia cell lines: K562; HL-60; RPMI8402. Human lung cancer cell line: A549; NCI-H23; NCI-H226; NCI-H522; NCI-H460; DMS273; DMS114. Human melanoma cell lines: LOX-IMVI. Human ovarian cancer cell lines: SK-OV-3; OVCAR-3; OVCAR-4; OVCAR-5; OVCAR-8. Human pancreatic cancer cell lines: BxPC-3; SW1990. Human renal cancer cell lines: 786-O; RXF-6312; ACHN.

## 4. Topo II Inhibitors Derived from Ascidians, Echinoderms and Marine Microalgae

### 4.1. Wakayin

**Figure 22:**
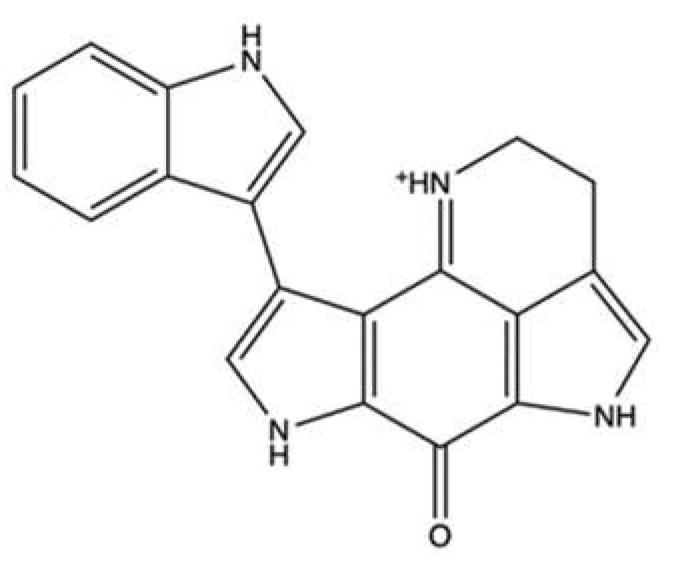
Chemical structure of wakayin (CAS number: 134781-25-2).

Wakayin (Figure 22) is a pyrroloiminoquinone alkaloid isolated from an ascidian, commonly called sea squirt, belonging to the species *Clavelina* [99]. 

In early studies evaluating its activity, wakayin induced cytotoxic effects on the human colon HCT-116 cancer cell line with an IC_50_ value of 0.5 μg/mL. On the same cell line, it inhibited topo II enzyme at a concentration of 250 μM [99]. Moreover, wakayin exhibited a higher cytotoxicity on DSBs repair-deficient CHO xrs-6 cells than on DSBs repair-proficient CHO BR1 cells. Their IC_50_ ratio was indeed 9.8, higher than that of ETO corresponding to 7.0. Those results clearly indicate DSB induction as a mechanism involved in the cytotoxicity of wakayin [100]. Taking into account this evidence and the planar quinonic structure of wakayin, it was hypothesized and then demonstrated that wakayin inhibited the decatenation of kDNA in a concentration-dependent manner in the range of 40 to 133 μg/mL [100]. However, the difference between the concentration inhibiting the purified enzyme (40–133 μg/mL) and the concentration exerting the cytotoxic effects (0.5 μg/mL) suggests that other mechanisms, not just topo II inhibition, could contribute to wakayin-induced DNA damage.

### 4.2. Ascididemin

**Figure 23:**
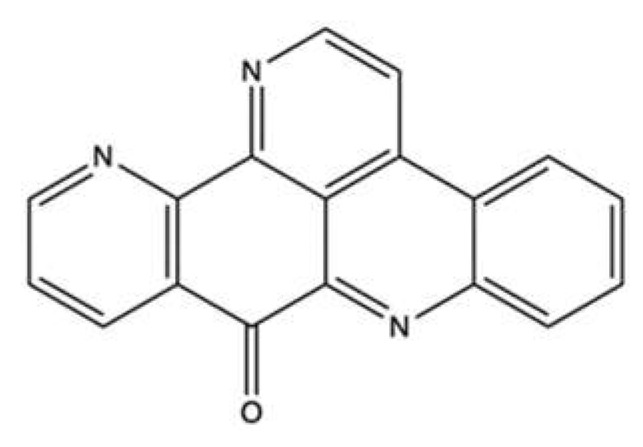
Chemical structure of ascididemin (CAS number: 114622-04-7).

Ascididemin (**ASC**, Figure 23) is a pyridoacridine alkaloid isolated from the mediterranean ascidian *Cystodytes dellechiajei* collected near the Balearic Islands [101] as well as from Okinawan ascidian *Didemnum* sp., from Kerama Islands [102].

It has been reported that **ASC** was 10-fold more cytotoxic in CHO xrs-6 (DSBs repair deficient) than in CHO BR1 (DSBs repair proficient) cells, while exhibiting identical toxicity in CHO-BR1 (SSB repair-proficient) and CHO-EM9 (SSB repair-deficient) cells, raising the hypothesis that DSBs were involved in its in vitro anticancer activity [103]. Moreover, **ASC** was cytotoxic on human leukemia, colon, and breast cancer cell lines [102]. Cytotoxicity elicited by **ASC** (Table 3) was related to the induction of Casp-dependent apoptosis, even at the lowest concentrations [102,104]. Meanwhile, it inhibited the growth of the non-malignant African green monkey kidney cell line BSC-1, revealing a lack of selectivity against cancer cells [103]. 

**ASC** was shown to inhibit topo II activity at a concentration equal to 30 μM [105]. Nearly 10 years later, Dassonneville and colleagues evaluated its interaction with topo II and demonstrated that this compound can (i) inhibit DNA ligation after it has been cleaved by topo II, and (ii) stimulate DNA cleavage with most cleavage sites having a C on the side of the cleaved bond [104]. Based on these results, **ASC** could be defined as a site-specific topo II poison for the purified enzyme, although its activity appeared to be inferior compared to the positive control ETO [104]. However, the capability of **ASC** to function as a topo II poison was not demonstrated in cellular assays. Indeed, comparing the cytotoxic activity of **ASC** on human leukemia cells sensitive (HL-60) or resistant (HL-60/MX2) to mitoxantrone, **ASC** was cytotoxic with similar IC_50_ values (0.48 μM for HL-60 and 0.65 μM for HL-60/MX2) [104]. Matsumoto and coworkers performed a cell-free assay to clarify the mechanism of action of **ASC**. The results proved that **ASC** was able to cleave the DNA in a concentration- and time-dependent manner, even in the absence of topo II. Moreover, experimental results demonstrated (i) the generation of ROS, (ii) that antioxidants treatment protected against DNA cleavage, and (iii) that cells deficient in ROS-induced damage repair system were more susceptible to **ASC**. On the whole, those results suggest that ROS production is involved in the cytotoxicity of **ASC** [106]. The production of ROS could be due to the direct reduction of **ASC** iminoquinone heterocyclic ring to semiquinone, with production of H_2_O_2_ [106]. Considering the potential of **ASC** to intercalate in DNA, it is probable that ROS production occurs in proximity of the nucleic acid, thereby producing DNA damage [106]. 

### 4.3. Gymnodinium *sp.* A_3_ Acidic Polysaccharide

The OKU-1 strain of the marine microalga, *Gymnodinium* sp. *A_3_* (**GA3**), was first discovered in the water of the Seto Inland Sea (Japan). **GA3** generates an extracellular acidic polysaccharide, which is a D-galactan sulfate associated with L-(+)-lactic acid, denominated **GA3P** [107]. 

Umemura and coworkers evaluated different **GA3P** formulations bearing high (>80%) and low (<20%) lactic acid percentage (**GA3Pl+** and **GA3Pl−**, respectively) [108]. Both preparations of **GA3P** inhibited kDNA decatenation with similar IC_50_ values (0.048 μg/mL for **GA3P+** and 0.052 μg/mL for **GA3P−**), proving that **GA3P** was a topo II inhibitor and that lactic acid percentage had no impact on topo II inhibition [108]. Gel electrophoresis of pT2GN plasmid DNA revealed that **GA3P+** did not induce the accumulation of cleavable complexes and acted as a catalytic inhibitor. Furthermore, the analysis of plasmid DNA showed that **GA3P+**, when simultaneously added to teniposide, inhibited the stabilization of teniposide-induced cleavable complexes [108]. 

In a large panel of cells, the polysaccharide slightly inhibited cell proliferation with GI_50_ values ranging from 0.67 to 11 μg/mL [108]. However, no further cellular assays were undertaken to elucidate the cytotoxic activity or the possible death mechanism exerted by the compound. Despite evidence showing that **GA3P+** was a topo II catalytic inhibitor, its chemical profile and high molecular weight can hamper its entry into the nucleus and its interaction with DNA or topo II. Certainly, further studies will be required to clarify the mechanism of action of **GA3P** against cancer cells. 

### 4.4. Echinoside A

**Figure 24:**
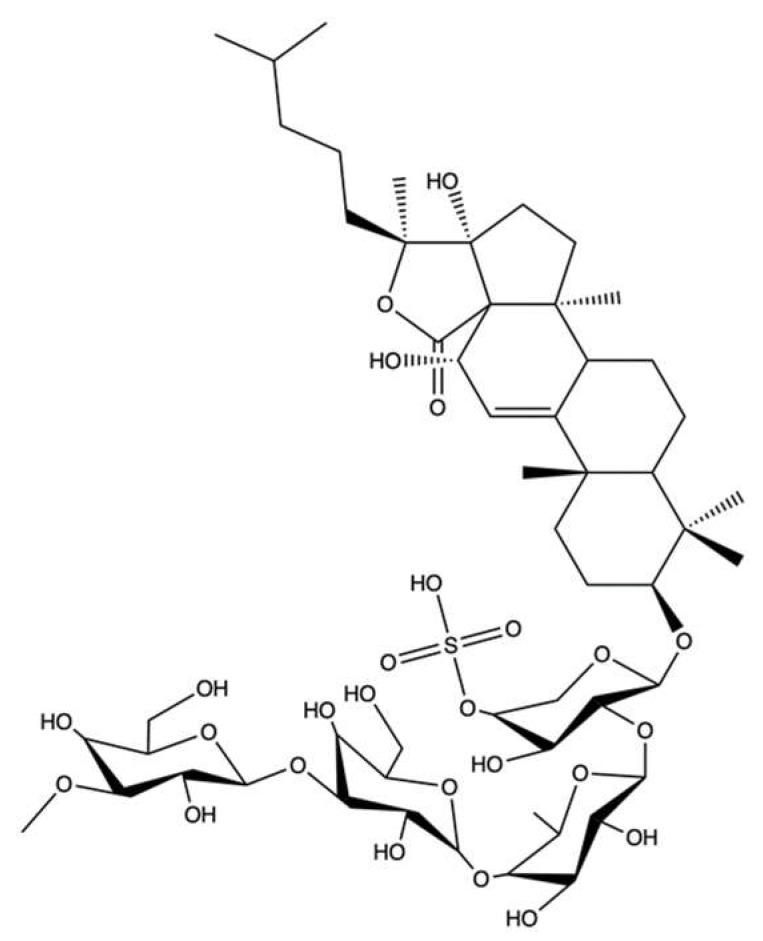
Chemical structure of echinoside A (CAS number: 75410-53-6).

Echinoside A (**ECH**, Figure 24) is a saponin isolated from the sea cucumber *Holothuria nobilis* (Selenka), an echinoderm retrieved from the sea ground of the Dongshan Island (P. R. China) [109].

**ECH** exerted a broad-spectrum anticancer activity against a panel of 26 human and murine cancer cell lines with very similar IC_50_ ranging from 1.0 to 6.0 μM [109]. Fluorescent TUNEL staining of **ECH**-treated HL-60 cells and DNA fragmentation indicated that the observed cytotoxicity resulted from Casp-dependent apoptosis. The potent effects observed in cancer cells were confirmed by in vivo experiments on animal cancer models (Table 3). 

An extensive and comprehensive set of in vitro experiments with topo IIα enzyme was conducted to investigate its topo II inhibitor activity. The results indicate that **ECH** effectively reduced the pBR322 plasmid DNA relaxation and suppressed kDNA decatenation [109]. An assay with top IIα extracted from HL-60 cells proved that **ECH** 0.5 μM induced the formation of stable cleavage complexes, which is a common mechanism for topo II poisons, along with intercalation in DNA. However, two different experiments (Table 3) reported that **ECH** was a non-intercalative agent, even at high concentrations [109]. The activity of **ECH** toward topo IIα-DNA binding was evaluated using a fluorescence anisotropy assay, which revealed that **ECH** inhibited the binding between the enzyme and DNA. Molecular docking studies clarified that **ECH**, through its sugar moiety, established strong hydrogen bonds with the DNA binding site of topo IIα, working as a catalytic inhibitor that competes with DNA for the substrate [109].

Further studies explored the effects of **ECH** on the cleavage/religation equilibrium using a cell-free assay. **ECH** produced an increase in DNA cleavage and enhanced DSBs formation, without significant effects on religation [109]. The ability of **ECH** to promote DNA cleavage without affecting DNA ligation makes it similar to topo II poisons such as ellipticin, genistein, and quinolones [110,111], which act with the same mechanism. However, **ECH** has been found to possess the peculiar characteristics of (i) blocking the noncovalent binding of topo IIα to DNA by competing with DNA for the DNA-binding domain of the enzyme, and (ii) hindering topo IIα-mediated pre-strand passage cleavage/religation equilibrium. Taken together, the studies presented above suggest that **ECH** is a potent non-intercalative topo II inhibitor with a peculiar mechanism of action. It acts as a topoisomerase poison (stabilization of cleavable complexes and induction of DSBs) and a catalytic inhibitor (inhibition on the topo II-DNA binding, interference with the pre-strand passage cleavage/religation equilibrium). Due to these characteristics, it constitutes a promising starting point for the development of anticancer drugs based on topo II inhibition 

### 4.5. Eusynstyelamide B

**Figure 25:**
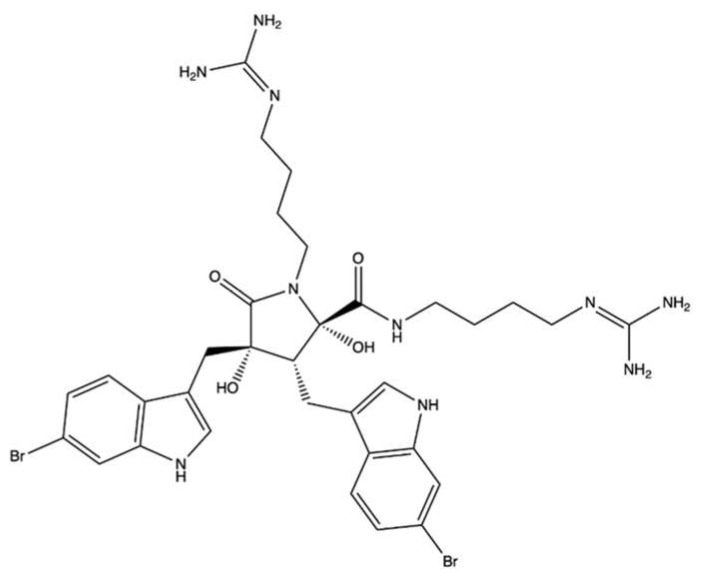
Chemical structure of eusynstyelamide B (CAS number: not available).

Eusynstyelamide B (**EUB**, Figure 25) is a bis-indole alkaloid extracted from the marine ascidian *Didemnum candidum* found in the Great Barrier Reef [112].

**EUB** was able to induce cytotoxicity in breast MDA-MB-231 and LNCaP prostate cancer cells [112,113]. Table 3 reports the differences in gene and protein expression between MDA-MB-231 and LNCaP cell lines, emphasizing the cell line-specific mechanisms of **EUB**. The COMET assay and the quantitative evaluation of γ-H2A.X foci supported the production of DNA damage via DSBs in both cell lines. 

With the aim to investigate whether the observed DNA damage derived from the direct interaction of **EUB** with DNA, a displacement assay and a DNA melting temperature analysis were performed. Both demonstrated that **EUB** did not directly interact with DNA but instead acted as a topo II poison [113]. **EUB** was also highly cytotoxic in two non-transformed cell lines (NFF primary human neonatal foreskin fibroblasts and RWPE-1 epithelial prostate cell line), with IC_50_ values even lower than that reported on tumor cell lines. NFF and RWPE-1 cells are highly proliferating and express high levels of topo IIα [114]. This means that the effects of **EUB** were not specific for cancer cells. Further in vitro and in vivo studies have to be performed to assess the safety profile of **EUB**.

**Table 3:** Topo II inhibitors derived from ascidians, echinoderms, and marine microalgae.

Compounds	Source	Topo II Inhibitory Activity			Antitumor Effect(s)			Ref
		Assay	Concentration(s) Tested or IC_50_	Outcomes	Experimental Model	Cytotoxic Activity	Other Antitumor Mechanism(s)	
Ascididemin	Ascidians *Cystodytes dellechiajei* and *Didemnum* sp.				L1210 ^d^	IC_50_ (time not indicated): 0.39 µg/mL	↑ Ca^2+^ release in sarcoplasmic reticulum	[102]
					P388 ^d^	IC_50_ (time not indicated): 0.4 µM		[103]
					HCT-116	IC_50_ (time not indicated): 0.3 µM		
					MCF-7	IC_50_ (time not indicated): 0.3 µM		
					CHO xrs-6 ^b^	IC_50_ (time not indicated): 0.03 µM		
					CHO EM9 ^c^	IC_50_ (time not indicated): 0.3 µM		
			IC_50_: 30 µM	↓ Topo II activity				[105]
		Cell free DNA relaxation assay using supercoiled pKMp27 DNA plasmid and topo II α	0.5–50 µM	Weak ↓ topo II activity	HL-60	IC_50_^a^ (72 h): 0.48 µM	Apoptosis (caspase-3 activation, PARP cleavage, DNA fragmentation) Cell cycle inhibition (↑ cells in sub-G1 phase, ↓ cells in G1 and G2 phases)	[104]
		Cell-free DNA cleavage assay using radiolabeled pKMp27 DNA plasmid	25–100 µM	Stimulation of DNA cleavage Stabilization of DNA–topoisomerase II covalent complexes	HL-60/MX2 ^e^	IC_50_^a^ (72 h): 0.65 µM	Cell cycle inhibition (↑ cells in sub-G1 phase, ↓ cells in G1 and G2 phases)	
		Cell-free DNA cleavage assay using radiolabeled and supercoiled rf M13 mp19 DNA	91 µM	↓ Topo II activity	CHO AA8	IC_50_ (72 h): 3.1 µM		[106]
					CHO EM9 ^c^	IC_50_ (72 h): 0.4 µM		
					CHO xrs-6 ^b^	IC_50_ (72 h): 0.7 µM		
					HCT-116	IC_50_ (72 h): 0.1 µM		
					KB	IC_50_ (72 h): 0.6 µM		
					P388 ^d^	IC_50_ (time not indicated): 0.4 µM		[115]
Bengacarboline	Ascidian *Didemnum* sp.		32 µM	↓ Topo II activity				[116]
					A-549	IC_50_ (72 h): 7.1 µM		[117]
					BxPC3	IC_50_ (72 h): 10.0 µM		
					LoVo	IC_50_ (72 h): 9.9 µM		
					MCF-7	IC_50_ (72 h): 8.6 µM		
Echinoside A	Echinoderm *Holothuria nobilis*	Cell-free decatenation reaction of kinetoplast DNA with topo IIα	1–125 µM	↓ DNA decatenation	K562	IC_50_ (time not indicated): 5.42 µM		[109]
					K562/A02 ^e^	IC_50_ (time not indicated): 5.31 µM		
					MCF-7	IC_50_ (time not indicated): 1.32 µM		
					MCF-7/ADR^e^	IC_50_ (time not indicated): 1.26 µM		
					KB	IC_50_ (time not indicated): 2.78 µM		
					KB/VCR^e^	IC_50_ (time not indicated): 3.29 µM		
		In vivo complexes of the enzyme (ICE) bioassay	0.5–1 μM	Formation of stable cleavage complexes	HL-60	Not indicated	Apoptosis (pro-caspase-3 and pro-caspase-8 cleavage, PARP degradation) DNA damage (↑ γ-H2AX protein expression)	
		Cell-free DNA relaxation assay using supercoiled pBR322 plasmid DNA and topo IIα	5–125 µM	↓ DNA relaxation	KM mice inoculated with S-180 ^d^ cells		↓ Tumor growth (1.5, 3.0 mg/kg/day for 7 days i.v.)	
		Cell-free DNA unwinding assay using supercoiled pBR322 plasmid DNA and topo IIα	5–125 µM	No intercalation in the DNA	KM mice inoculated with H22 ^d^ cells		↓ Tumor growth (1.5, 3.0, 6.0 mg/kg/day for 7 days i.v)	
		Ethidium bromide displacement fluorescence assay with human topo IIα	1–125 µM	No intercalation in the DNA	Nude mice bearing human prostate carcinoma PC-3 xenografts		↓ Tumor growth (2.6, 5.2 mg/kg/week for 4 weeks i.v.)	
		Cell-free topo IIα –mediated DNA religation assay using supercoiled pBR322 plasmid DNA		↑ Topo IIα-dependent DSBs formation				
		Cell free ATP hydrolysis assay with human topo IIα assay	100 µM	No alteration of the ATPase activity of topo IIα	Nude mice bearing human prostate carcinoma PC-3 xenografts		↓ Tumor growth (2.6, 5.2 mg/kg/week for 4 weeks i.v.)	
Eusynstyelamide B	Ascidian *Didemnum candidum*				MDA-MB-231	IC_50_ (72 h): 4.95 µM	Apoptosis (PARP cleavage) Cell cycle inhibition (↑ cells in G2/M phase, ↓ cells in G0/G1 phase)	[112]
					LNCaP	IC_50_ (72 h): 5.0 µM	Cell cycle inhibition (↑ cells in G2/M phase, ↓ S phase progression, ↓ CDK1, CCNB1, ↓ CDC25A, ↓ CDC2 gene expression, ↑ CDKN1A gene expression, ↑ p21^CIP1/WAF1^, ↓ total CDC2 protein expression DNA damage induction (↑ γH2AX foci, ↓ expression of MKI67, ↑ GADD45A, GADD45G ↑ CHK2 phosphorylation	[113]
		Ethidium bromide displacement fluorescence assay with topo II	6.25–50 µM	No intercalation	MDA-MB-231		Cell cycle inhibition (↑ cells in G2/M phase, ↓ cells in G0/G1 phase, ↑ CDKN1A gene expression ↑ p21^CIP1/WAF1^ ↓ CCNB1 gene expression, ↑ total CDC2 protein, ↑ p53, ↑ total p53 expression) DNA damage (↑ γH2AX foci, ↑ CHK2 phosphorylation ↑ CHK1 phosphorylation)	
		DNA melting temperature analysis	6.25–50 µM	No direct interaction with DNA				
		Decatenation reaction of kinetoplast DNA with topo II	25–100 µM	↓ Decatenation of kDNA				
GA3Pl+	Microalga *Gymnodinium* sp. A_3_	Cell-free decatenation reaction of kinetoplast DNA with recombinant human topo IIα	IC_50_: 0.017 μg/mL	↓ Topo IIα activity	HBC-4	GI_50_ (time not indicated): 5.2 µg/mL		[108]
		Cell-free DNA cleavage assay using supercoiled pT2GN plasmid DNA and recombinant human topo IIα	IC_50_ 0.048 μg/mL	↓ Topo IIα activity	BSY-1	GI_50_ (time not indicated): 0.67 µg/mL		
					HBC-5	GI_50_ (time not indicated): 6.2 µg/mL		
					MCF-7	GI_50_ (time not indicated): 2.9 µg/mL		
					MDA-MB-231	GI_50_ (time not indicated): 1.5 µg/mL		
					U251	GI_50_ (time not indicated): 2.0 µg/mL		
					SF-268	GI_50_ (time not indicated): 4.7 µg/mL		
					SF-295	GI_50_ (time not indicated): 2.7 µg/mL		
					SF-539	GI_50_ (time not indicated): 1.8 µg/mL		
					SNB-75	GI_50_ (time not indicated): 1.5 µg/mL		
					SNB-78	GI_50_ (time not indicated): 2.4 µg/mL		
					HCC2998	GI_50_ (time not indicated): 2.3 µg/mL		
					KM-12	GI_50_ (time not indicated): 3.7 µg/mL		
					HT-29	GI_50_ (time not indicated): 3.6 µg/mL		
					WiDr	GI_50_ (time not indicated): 3.1 µg/mL		
					HCT-15	GI_50_ (time not indicated): 3.4 µg/mL		
					HCT-116	GI_50_ (time not indicated): 3.7 µg/mL		
					NCI-H23	GI_50_ (time not indicated): 2.8 µg/mL		
					NCI-H226	GI_50_ (time not indicated): 2.2 µg/mL		
					NCI-H522	GI_50_ (time not indicated): 1.3 µg/mL		
					NCI-H460	GI_50_ (time not indicated): 3.8 µg/mL		
					A-549	GI_50_ (time not indicated): 11 µg/mL		
					DMS273	GI_50_ (time not indicated): 2.0 µg/mL		
					DMS114	GI_50_ (time not indicated): 2.7 µg/mL		
					LOX-IMVI	GI_50_ (time not indicated): 6.3 µg/mL		
					OVCAR-3	GI_50_ (time not indicated): 2.2 µg/mL		
					OVCAR-4	GI_50_ (time not indicated): 3.2 µg/mL		
					OVCAR-5	GI_50_ (time not indicated): 6.8 µg/mL		
					OVCAR-8	GI_50_ (time not indicated): 4.1 µg/mL		
					SK-OV-3	GI_50_ (time not indicated): 8.1 µg/mL		
					RXF-631L	GI_50_ (time not indicated): 9.1 µg/mL		
					ACHN	GI_50_ (time not indicated): 8.3 µg/mL		
					St-4	GI_50_ (time not indicated): 8.4 µg/mL		
					MKN1	GI_50_ (time not indicated): 3.0 µg/mL		
					MKN7	GI_50_ (time not indicated): 5.9 µg/mL		
					MKN28	GI_50_ (time not indicated): 7.0 µg/mL		
					MKN45	GI_50_ (time not indicated) 2.9 µg/mL		
					MKN74	GI_50_ (time not indicated): 4.6 µg/mL		
GA3Pl-	Microalga *Gymnodinium* sp. A_3_	Decatenation reaction of kinetoplast DNA with recombinant human topo IIα	IC_50_ 0.015 μg/mL	↓ Topo IIα activity				
		Cell-free DNA cleavage assay using supercoiled pT2GN plasmid DNA and recombinant human topo IIα	IC_50_ 0.052 μg/mL					
Meridine	Ascidian *Amphicarpa meridiana*	Decatenation reaction of kinetoplast DNA	IC_50_ 3 µM	↓ Topo II activity	P388 ^d^	IC_50_ (72 h): 0.08 µM		[118]
					A-549	IC_50_ (72 h): 0.08 µM		
					HT-29	IC_50_ (72 h): 0.84 µM		
					MEL-28	IC_50_ (72 h): 0.08 µM		
			75 µM	↓ Topo II activity				[105]
Wakayin	Ascidian *Clavelina* sp.				HCT-116	IC_50_ (time not indicated): 0.5 µg/mL		[99]
		Decatenation reaction of kinetoplast DNA with avian topo II	40–133 µg/mL	↓ Topo II activity	CHO BR1	IC_50_ (72 h): 3.05 µg/mL		[100]
					CHO xrs-6 ^b^	IC_50_ (72 h): 0.31 µg/mL		

↑: upregulation/induction; ↓: downregulation/inhibition; ^a^: concentration that inhibits 50% of the proliferation ^b^: DNA-double strand break repair-deficient cells; ^c^: DNA-single strand break repair-deficient cells; ^d^: murine cancer cells; ^e^: multidrug resistant cancer cells; Ca^2+^: calcium; CCBN1: cyclin B1; CDC2: cell division cycle 2; CDC25A: cell division cycle 25 homolog A; CDK1: cyclin dependent kinase 1; CDKN1A: cyclin dependent kinase inhibitor 1A; CHK1: checkpoint kinase 1; CHK2: checkpoint kinase 2; CHO: Chinese hamster ovary; DSBs: double-strand breaks; γ-H2AX: phosphorylated H2A histone family member X; GADD45A: growth arrest and DNA damage inducible alpha; GADD45G: growth arrest and DNA damage inducible gamma; GI_50_ concentration that inhibits 50% of cell growth; IC_50_: concentration that inhibits 50% of the investigated activity; i.v.: intravenous; MKI67: marker of proliferation Ki-67; p21^CIP1/WAF1^: cyclin dependent kinase inhibitor 1; PARP: poly (ADP-ribose) polymerase. Human breast cancer cell lines: MCF-7; MDA-MB-231; BSY-1. Human colon cancer cell lines: HCT-116; HT-29; HCT2998; KM-12; WiDr; HCT-15. Human gastric cancer cell line: MKN45; St-4; MKN1; MKN7; MKN28; MKN74. Human glioma cell lines: U251. Human glioblastoma cell lines: SF-268; SF-295; SF-539; SNB-75; SNB-78. Human leukemia cell lines: K562; HL-60; Human lung cancer cell line: A549; NCI-H23; NCI-H226; NCI-H522; NCI-H460; DMS273; DMS114. Human melanoma cell lines: LOX-IMVI; MEL-28. Human ovarian cancer cell lines: SK-OV-3; OVCAR-3; OVCAR-4; OVCAR-5; OVCAR-8. Human pancreatic cancer cell lines: BxPC-3. Human renal cancer cell lines: RXF-6312; ACHN. Human prostate cancer cell lines: PC-3; LNCaP.

## 5. Conclusions

Of the compounds discussed in this review, only a few acts as topo II poisons (adociaquinone B and **EUB**) and as catalytic inhibitors (**neo** and **apl-1**). Several others exhibit topo II inhibitory activity but, due to the paucity of experimental evidence, their mode of inhibition has not been elucidated, making it difficult to establish their mechanism of action. 

Although topo II inhibitors, particularly topo II poisons, are successfully used as anticancer agents, the occurrence of drug resistance and severe side effects, such as cardiotoxicity and the development of secondary malignancies, limit their use [43]. An approach to overcome these limitations could be the use of dual inhibitors. Multiple marine-derived compounds described in this review such as 25-acetals manoalide, xestoquinone, **HA-A**, and **M7**, inhibit both topo I and topo II [55,60,61,76], while for others, topo II inhibitory activity is accompanied by the inhibition of Hsp90 [36,62,74] or HDAC [75,76]. The resulting advantages are manifold. Simultaneous inhibition of topo I and topo II could reduce the possible onset of resistance. The same advantage can be achieved by inhibiting topo II and Hsp90 [43]. Concerning topo II and HDAC inhibition, HDAC inhibition-mediated histone hyperacetylation increases chromatin decondensation and DNA accessibility. These effects may promote topo II binding and enhance topo II inhibiting activity [43]. Among the marine compounds presented in this review, heteronemin is the most interesting. Indeed, its cytotoxic activity was highly multimechanistic, with inhibition of the catalytic activities of both topo I and topo II and inhibition of Hsp90, associated with oxidative and ER stress. However, the dual inhibitors are often compounds with a high molecular weight [119], which could limit their druggability and their safety profile as well as indicate that their pharmacokinetics should be thoroughly explored

Another issue to consider is the ability of topo II inhibitors to cause DNA lesions that, if not repaired or not cytotoxic, could lead to chromosome aberrations and secondary malignancies such as leukemias [120]. Although topo II catalytic inhibitors are usually associated with no or limited direct DNA damage [121], some marine-derived topo II catalytic inhibitors presented in this review induce DNA DSBs and/or increase the protein expression of DNA damage-related proteins. Thus, it would be of great relevance to clarify whether their genotoxicity results from their topo II catalytic inhibition or involves different mechanisms. A further concern related to the toxicological profile is the lack of selectivity toward cancer cells exhibited by some marine compounds, which prompts more extensive studies on non-transformed cells to assess the safety of such molecules. 

Lastly, some marine compounds exhibited a strong binding affinity for topo II, demonstrated through molecular docking studies. Among those, the most interesting are **neo**, **ECH**, and sulochrin, which are characterized by a binding energy of -61.8, -39.21, and -12.11 kcal/mol, respectively. However, in some cases, this interaction has not been confirmed by cellular assays, making it difficult to know whether topo II binding leads to the actual inhibition of the enzyme activity. Thus, at least DNA decatenation and/or relaxation assays are necessary to confirm their topo II inhibitory activity. These cell-free assays certainly provide early indications of the effective inhibition of topo II. However, they may not be sufficient because, as shown for secoadociaquinone A and B and **GA3P** [77,108], their inhibitory activity on the purified enzyme does not necessarily lead to the inhibition of topo II at the cellular level.

In conclusion, in this review, we reported current studies on marine-derived compounds targeting topo II, highlighted their pharmacological potential, and discussed their toxicological issues.

## Data Availability

Not applicable.
